# c-Myc/GRPEL1 maintains fatty acid synthesis via FASN to support PDAC cell proliferation

**DOI:** 10.1038/s41419-026-08439-0

**Published:** 2026-02-05

**Authors:** Jing Wang, Liyuan Zhang, Keke Chen, Fangze Wei, Wendi Li, Chanjuan Cui, Feng Chen, Bing Wei, Tao Huang, Hezhi Fang, Wei Cui

**Affiliations:** 1 Department of Clinical Laboratory, State Key Laboratory of Molecular Oncology, National Cancer Center/National Clinical Research Center for Cancer/Cancer Hospital, Chinese Academy of Medical Sciences and Peking Union Medical College, Beijing, China; 2 Zhejiang Provincial Key Laboratory of Medical Genetics, College of Laboratory Medicine and Life Sciences, Wenzhou Medical University, Wenzhou, China; 3 The Affiliated Cancer Hospital of Zhengzhou University & Henan Cancer Hospital, Zhengzhou, China

**Keywords:** Cancer metabolism, Oncogenes

## Abstract

Pancreatic ductal adenocarcinoma (PDAC) cells undergo mitochondrial metabolic reprogramming to support their proliferation. However, the mechanisms by which mitochondrial protein quality control (MPQC) regulates cell metabolism remain unclear. Here, we found that c-Myc promotes PDAC cell proliferation by transcriptionally upregulating the expression of GRPEL1, an essential MPQC component. Mechanistically, c-Myc-regulated GRPEL1 maintains oxidative phosphorylation (OXPHOS) and minimizes ROS accumulation, thereby facilitating de novo fatty acid (FA) synthesis through the transcriptional upregulation of fatty acid synthase (FASN) expression. Targeting the c-Myc/GRPEL1 axis to block FASN-regulated FA synthesis inhibited PDAC cell proliferation and tumor growth in both cell models and patient-derived organoids (PDOs), whereas FA supplementation partially reversed this inhibitory effect. Clinically, c-Myc expression is positively associated with the levels of MPQC components in pancreatic ductal cells, with GRPEL1 ranking among the top hits. Furthermore, c-Myc, GRPEL1, and FASN are all expressed at higher levels in PDAC tissues than in peri-tumoral pancreatic tissues, and both c-Myc and GRPEL1 expression levels are positively correlated with that of FASN. These findings suggest that therapeutic inhibition of FA synthesis may be promising for treating PDAC patients with active c-Myc/GRPEL1/FASN signaling. Overall, this study demonstrates that FA synthesis mediated by the c-Myc/GRPEL1/FASN axis is essential for PDAC growth.

## Introduction

Pancreatic cancer is a highly malignant tumor of the digestive system. It primarily results from the malignant transformation of ductal epithelial cells [1–3]. Approximately 90% of pancreatic cancer cases are pancreatic ductal adenocarcinoma (PDAC), which is characterized by late detection, frequent metastasis, and a poor prognosis [4]. Currently, surgical intervention is often not feasible for advanced cases, and the efficacy of radiotherapy and chemotherapy is limited. As a result, the 5-year survival rate for PDAC is below 11% [5–8]. These challenges underscore the necessity to explore the complex molecular mechanisms of PDAC development and to devise new therapeutic strategies for this disease.

Metabolic reprogramming allows pancreatic cancer to flourish under conditions of nutrient deprivation and hypoxia [9, 10]. This process is a key factor in its growth, progression, and resistance to therapy [11]. Mitochondria, serving as cellular “powerhouses,” play a pivotal role in regulating glucose, lipid, and amino acid metabolism [12]. Their normal function relies on the homeostasis of their internal proteins. Normally, proteins encoded by nuclear genes are delivered to mitochondria through highly selective mechanisms, including molecular chaperones, the mitochondrial unfolded protein response (mtUPR), and proteases, to ensure proper protein import, targeting, and folding [13]. This process, also known as mitochondrial protein quality control (MPQC), is required to maintain mitochondrial protein homeostasis and functional. Activation of MPQC can regulate mitochondrial biogenesis and protein expression at the organellar and molecular levels, respectively, thereby affecting cell metabolism and determining cell fate [14–16]. Studies have shown that quality control proteases, such as LonP1 and ClpP, recognize and degrade misfolded or damaged proteins, ensuring the activity of enzyme complexes involved in energy metabolism and providing stable ATP for continuously proliferating tumor cells [17]. Mitochondria also synthesize cofactors, such as iron-sulfur clusters, to stabilize the expression of metabolic enzymes or regulate their activity [18]. In addition, mitochondria produce metabolic intermediates such as acetyl-CoA, succinate, α-ketoglutarate (α-KG), and fumarate, which can mediate the metabolic state of the whole organism by acting as signaling molecules or by altering epigenetic modifications in the genome to regulate gene expression [19]. Recent studies have demonstrated the effectiveness and feasibility of targeting mitochondrial metabolism for cancer therapy. For example, depletion of IscU2 disrupts the synthesis of Fe-S clusters, leading to excess α-KG in PDAC cells, changing the intracellular methylation level, and ultimately achieving anti-tumor effects [20]. However, the relationship between dysregulation of MPQC-related proteins, alterations in mitochondrial metabolic function, and tumorigenesis, particularly in PDAC, remains largely unknown.

Using bioinformatics analysis, we identified GRPEL1 as a target with potential clinical relevance. We characterized the regulatory role and mechanism of GRPEL1 in PDAC using a series of in vitro and in vivo experiments. Our results demonstrate that c-Myc acts as a transcription factor directly activating GRPEL1 expression in PDAC. Mechanistically, GRPEL1 deficiency suppresses the expression of FASN—a key enzyme in fatty acid synthesis—in a ROS-dependent manner, thereby impeding intracellular fatty acid synthesis and ultimately inhibiting PDAC tumor growth.

## Results

### *c-Myc* transcriptionally upregulates *GRPEL1* expression in PDAC cells

**Fig. 1:**
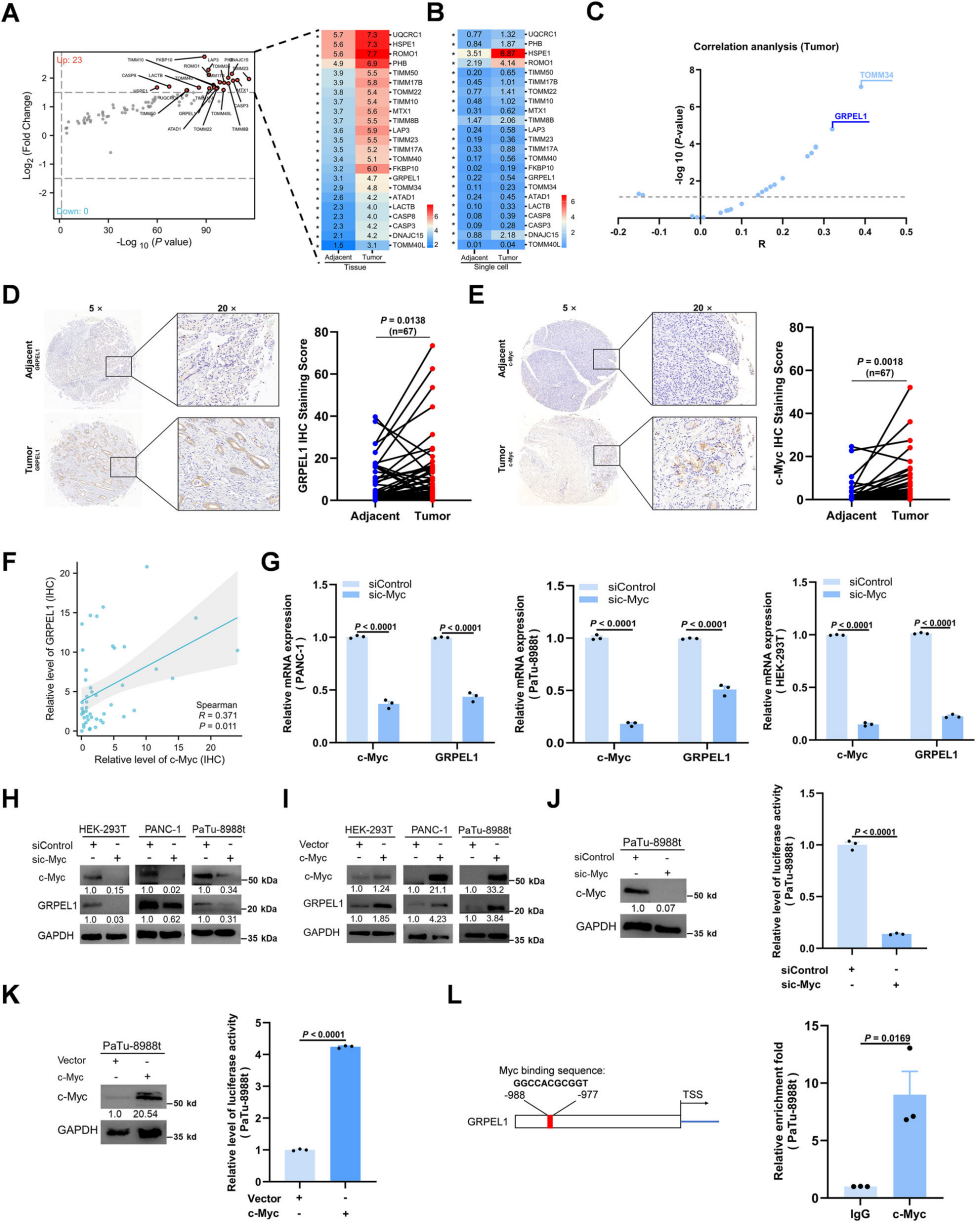
*c-Myc* transcriptionally upregulates *GRPEL1* expression in PDAC cells. **A** Volcano plot of the 86 MPQC genes in PDAC tumor tissues (TCGA, *n* = 179) and peri-tumoral pancreatic tissues (GTEx, *n* = 171). The upregulated genes are marked in red; the downregulated genes are marked in blue (|Log_2_FC| ≥ 1.5 & *P* < 0.05). The heat maps show the mRNA levels of the 23 significant DEGs. *, statistically significant, analyzed by one-way ANOVA. **B** Comparison of mRNA levels of 23 DEGs in normal and tumor pancreatic ductal cells at the single cell level (GEO accession number: GSE93326). *, statistically significant, analyzed by one-way ANOVA. **C** Correlation analysis of *c-Myc* and 23 DEGs in PDAC tissues (TCGA, *n* = 179). Representative IHC-staining images (left panel) and IHC scores (right panel) for GRPEL1 (**D**) and c-Myc (**E**) in 67 paired PDAC tissues. **F** Co-expression analyses of c-Myc and GRPEL1 in local PDAC tissues. **G** mRNA expression analysis of *c-Myc* and *GRPEL1* in PANC-1, PaTu-8988t, and HEK-293T cells transfected with a control siRNA or *c-Myc* siRNA. Immunoblotting analysis of c-Myc and GRPEL1 in HEK-293T, PANC-1 and PaTu-8988t cells transfected with a control siRNA or *c-Myc s*iRNA (**H**), or in cells with or without *c-Myc* overexpression (**I**). GAPDH was used as an internal control. **J**, **K** Luciferase reporter assay in PaTu-8988t cells with modulated c-Myc expression. Left panels: immunoblotting analysis of c-Myc upon depletion (**J**) or overexpression (**K**). GAPDH was used as a loading control. **L** ChIP-qPCR analyses were performed with an anti-IgG or an anti-c-Myc antibody in PaTu-8988t cells. The c-Myc binding sites on GRPEL1 promoters were predicted by the JASPAR database (https://jaspar2020.genereg.net) (left panel). qPCR analyses were performed with primers targeting the region from −1500 bp to the transcription start site (TSS) of the GRPEL1 promoter. The red box indicates the predicted c-Myc binding site. Data are presented as means ± SEM for bar graphs from at least three independent experiments. Representative images from three independent biological replicates are shown.

To investigate whether MPQC is associated with PDAC, we analyzed the expression levels of 86 previously characterized MPQC genes [13, 21, 22]. The expression of 23 of these genes was significantly upregulated (|Log_2_FC| ≥ 1.5 & *P* < 0.05) in PDAC tumors (TCGA) compared with peri-tumoral pancreatic tissues (GTEx) (Fig. 1A). This upregulation was confirmed in tumor ductal cells by analyzing a public single-cell sequencing dataset of PDAC (Fig. 1B). Collectively, these data suggest that enhanced MPQC is associated with PDAC.

It is well known that c-Myc is required for PQC activation in the endoplasmic reticulum to cope with accelerated protein turnover in cancer cells [23]. Mitochondria are the only other organelles containing a PQC system in mammalian cells; however, whether and how c-Myc regulates MPQC remains largely unknown. Co-expression analysis confirmed that *c-Myc* expression is positively associated with MPQC genes, including *GRPEL1* and *TOMM34* in tumors, and with *GRPEL1*, *TIMM50*, *TIMM17B*, *TIMM8B*, and *TOMM37* in peri-tumoral pancreatic tissues. Among these, GRPEL1 was the top-ranked c-Myc-associated MPQC component in both the TCGA and GTEx datasets (Figs. 1C and S1A). Both *GRPEL1* and *c-Myc* are associated with poor PDAC prognosis (Fig. S1B, C). Moreover, co-expression between GRPEL1 and c-Myc was further confirmed at the protein level in our local PDAC cohort (Fig. 1D–F). Depletion of c-Myc in two PDAC cell lines and in HEK-293T cells decreased GRPEL1 expression at both the mRNA and protein levels (Fig. 1G, H). Conversely, c-Myc overexpression upregulated GRPEL1 and its mRNA level (Figs. 1I and S1D). These results indicate that c-Myc promotes the expression of GRPEL1, raising the possibility that c-Myc influences MPQC by regulating GRPEL1.

To determine how c-Myc regulates GRPEL1 expression, we performed a luciferase reporter assay, which confirmed that c-Myc transcriptionally upregulates GRPEL1 (Fig. 1J, K). A ChIP assay further revealed that c-Myc directly binds to the region from −988 to −977 bp of the GRPEL1 promoter (Fig. 1L). Collectively, these data demonstrate that c-Myc serves as a transcription factor that directly regulates GRPEL1.

### c-Myc-regulated GRPEL1 supports PDAC cell proliferation

**Fig. 2:**
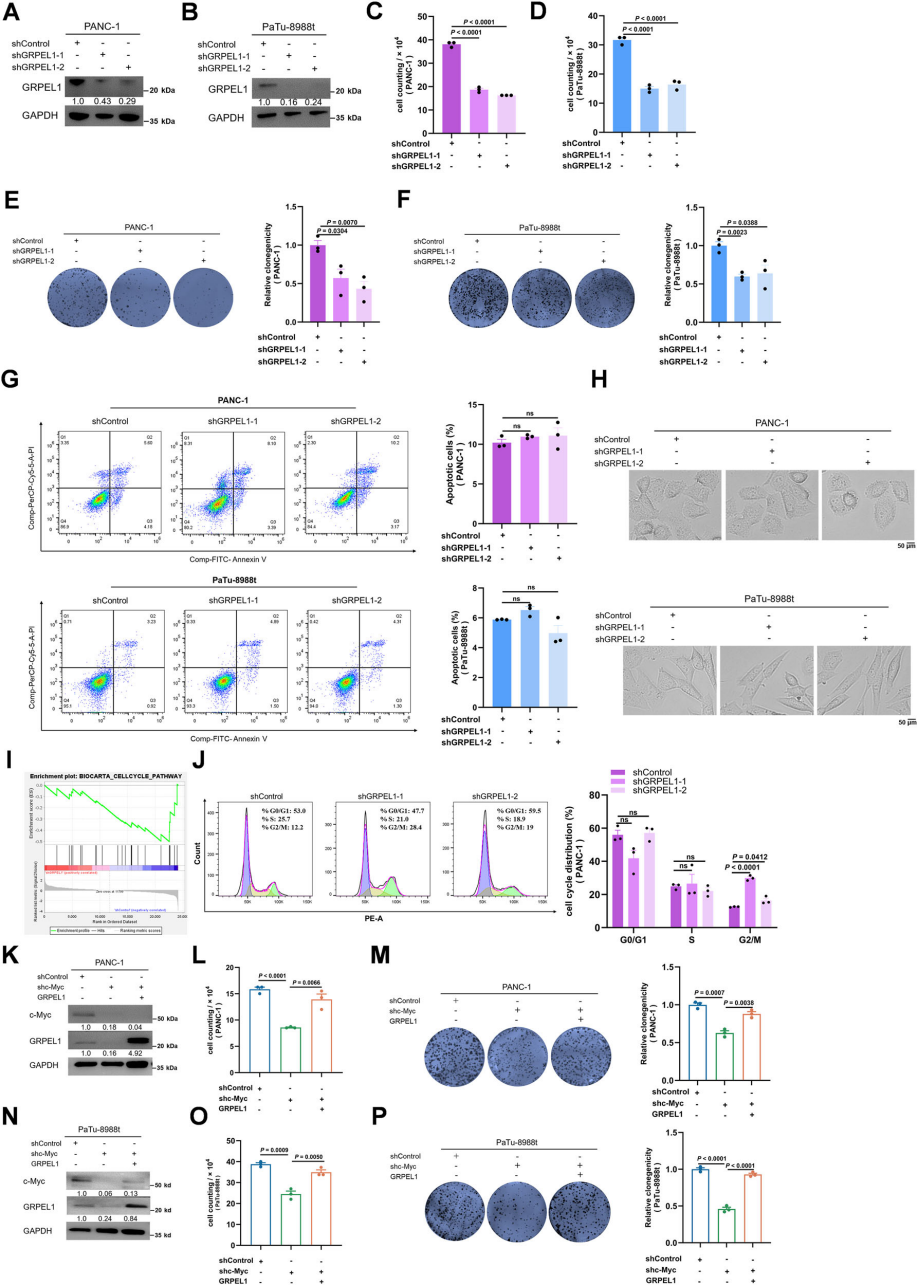
c-Myc-regulated GRPEL1 supports PDAC cell proliferation. **A**, **B** Immunoblot analysis of GRPEL1 protein expression level upon GRPEL1 depletion in PANC-1 and PaTu-8988t cells. **C**, **D** Proliferation of PANC-1 and PaTu-8988t cells upon GRPEL1 depletion. **E**, **F** Colony formation assay was performed to examine long-term growth of PANC-1 and PaTu-8988t cells upon GRPEL1 depletion. **G** Annexin-V & Dead Cell (7-AAD) flow cytometry analysis of PANC-1 and PaTu-8988t cells upon GRPEL1 depletion. The right panel shows the quantification of annexin V-positive cells in the left panels. **H** Representative images of cell morphology for PANC-1 and PaTu-8988t cells with or without GRPEL1 depletion. Scale bar = 50 μm. **I** GSEA of the cell cycle pathway using the BIOCARTA database in PaTu-8988t cells upon GRPEL1 depletion (*n* = 3). **J** Cell cycle of PANC-1 cells upon GRPEL1 depletion depleted was determined by flow cytometry. **K**–**P** Immunoblotting analysis with the indicated antibodies (**K**, **N**). Proliferation of PANC-1 (upper panels) and PaTu-8988t (lower panels) cells was evaluated by cell counting (**L**, **O**) and colony formation (**M**, **P**) assays. Data are presented as means ± SEM for bar graphs from at least three independent experiments. Representative images from three independent biological replicates are shown.

To investigate the contribution of GRPEL1 to PDAC, we depleted GRPEL1 (Fig. 2A, B) or overexpressed it (Fig. S2A, B) in two PDAC cell lines. Depletion of GRPEL1 resulted in decreased cell numbers (Fig. 2C, D) and colony formation (Fig. 2E, F) in both PANC-1 and PaTu-8988t cells, whereas GRPEL1 overexpression had the opposite effect (Fig. S2C–G). Unlike a previous report that GRPEL1 depletion induces apoptosis in ovarian cancer cells [24], depleting GRPEL1 neither stimulates apoptosis nor changes cellular morphology in these PDAC cell lines (Fig. 2G, H); however, suppression of the cell cycle, a cell proliferation-related pathway, was observed in GRPEL1-depleted PaTu-8988t cells (Fig. 2I). We experimentally confirmed a G2/M phase arrest in these cells (Figs. 2J and S2H). Moreover, overexpressing GRPEL1 partially restored the c-Myc depletion-induced arrest of cell proliferation and colony formation in both PANC-1 (Fig. 2K–M) and PaTu-8988t cells (Fig. 2N–P). Together, these results indicate that c-Myc-regulated expression of GRPEL1 is required for accelerated PDAC cell proliferation.

### GRPEL1 deficiency down-regulates FASN expression to inhibit cell proliferation

**Fig. 3:**
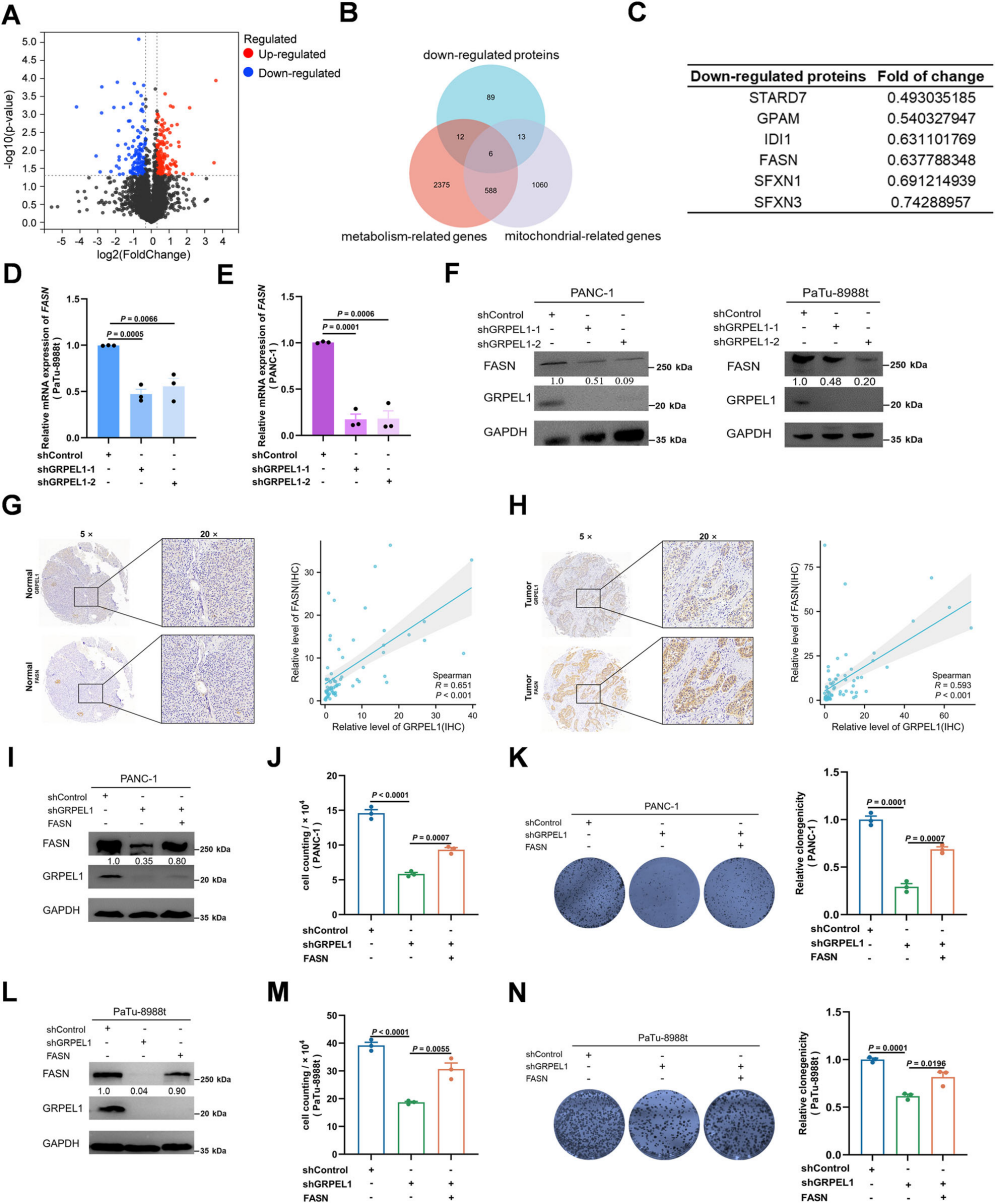
GRPEL1 deficiency down-regulates FASN expression to inhibit cell proliferation. **A** Differences in protein expression at the translational level were observed in PaTu-8988t cells with or without GRPEL1 depleted (upregulated with FC ≥ 1.25, downregulated with FC ≤ 0.75, and *P* < 0.05). **B** The Venn diagram shows the overlap among downregulated proteins, mitochondrial-related genes, and metabolism-related genes. **C** The six overlapping genes were STARD7, GPAM, IDI1, FASN, and SFXN1/3. **D**, **E** qPCR analyses of the relative mRNA levels of *FASN* in PaTu-8988t and PANC-1 cells upon GRPEL1 depletion. **F** Immunoblot analysis of FASN protein expression in PANC-1 and PaTu-8988t cells upon GRPEL1 depletion. **G** Representative IHC images (left panel) and co-expression analysis (right panel) of GRPEL1 and FASN in peri-tumoral pancreatic tissues. **H** Representative IHC images (left panel) and co-expression analysis (right panel) of GRPEL1 and FASN in PDAC tissues. **I**–**N** Immunoblotting analysis with the indicated antibodies (**I**, **L**). Proliferation of PANC-1 (upper panels) and PaTu-8988t (lower panels) cells was evaluated by cell counting (**J**, **M**) and colony formation (**K**, **N**) assays. Data are presented as means ± SEM for bar graphs from at least three independent experiments. Representative images from three independent biological replicates are shown.

In addition to mitochondrial protein import, folding, and stabilization, MPQC can also coordinate with the mitochondrial integrated stress response and retrograde signaling response for unconventional mitochondrial surveillance [25, 26]. To identify downstream targets of GRPEL1 and elucidate its regulatory mechanism in PDAC cell proliferation, we performed quantitative proteomics in PaTu-8988t cells with or without GRPEL1 depletion. A total of 172 upregulated and 120 downregulated proteins were identified (upregulated with FC ≥ 1.25, downregulated with FC ≤ 0.75, and *P* < 0.05) (Fig. 3A). Among these, six mitochondrial-localized metabolic proteins were downregulated in GRPEL1-depleted PaTu-8988t cells (Fig. 3B), including STARD7, GPAM, IDI1, FASN, and SFXN1/3 (Fig. 3C). Notably, only the mRNA level of *FASN* was downregulated due to *GRPEL1* depletion in PaTu-8988t cells (Figs. 3D and S3A), a finding that was confirmed in PANC-1 cells (Fig. 3E), indicating an unconventional MPQC role of GRPEL1 in regulating FASN expression. We examined the expression of MPQC-related markers ATF4 and p-eIF2α [13] and found that GRPEL1 deficiency did not affect their expression in PDAC cells, suggesting a non-MPQC mechanism for GRPEL1 regulation of FASN expression (Fig. S3B). The significant alteration of ATF4 and eIF2α expression upon c-Myc deficiency, which contrasts with the lack of effect from GRPEL1 depletion, highlights the diversity of c-Myc’s downstream targets and its multifaceted regulatory roles (Fig. S3C). Correspondingly, GRPEL1 depletion reduced, while its overexpression enhanced, FASN protein levels in both PANC-1 and PaTu-8988t cells (Figs. 3F and S3D). It was also noticed that GRPEL1 is positively associated with FASN expression in peri-tumoral pancreatic tissues and tumor pancreases, at both mRNA (Fig. S3E, F) and protein (Fig. 3G, H) levels. A positive association between *FASN* and *c-Myc* was also observed in cancerous and peri-tumoral pancreatic tissues (Fig. S3G, H). Given that FASN overexpression partially restored the FASN level, cell proliferation, and colony formation in GRPEL1-depleted PANC-1 (Fig. 3I–K) and PaTu-8988t cells (Fig. 3L–N), and that it similarly partially ameliorated the slower proliferation of PaTu-8988t cells caused by c-Myc deficiency (Fig. S3I, J), these findings suggest that c-Myc/GRPEL1-regulated FASN expression contributes to cell proliferation.

### GRPEL1 defects cause ROS accumulation, thereby inhibiting FASN expression

**Fig. 4:**
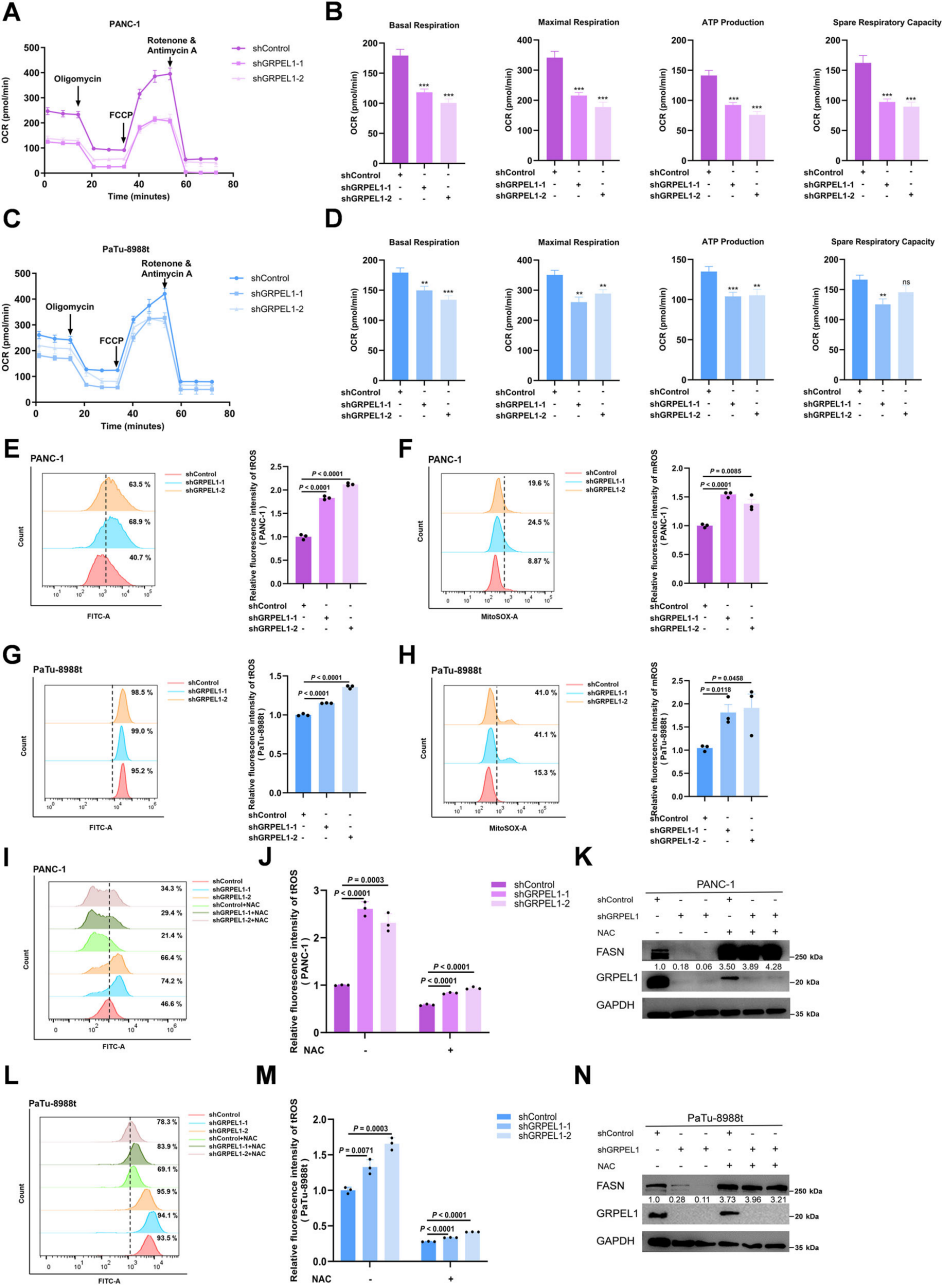
GRPEL1 defects cause ROS accumulation, thereby inhibiting FASN expression. **A**–**D** Seahorse analysis of OCR in PANC-1 (**A**) and PaTu-8988t (**C**) cells upon GRPEL1 depletion. The calculated parameters for basal respiration, maximal respiration, ATP production, and spare respiratory capacity are shown in (**B**) and (**D**). Flow cytometric analysis of cellular and mitochondrial ROS in PANC-1 (**E**, **F**) and PaTu-8988t (**G**, **H**) cells. Cells were stained with DCFH-DA (for cellular ROS) and MitoSOX Red (for mitochondrial ROS), respectively. Left panels: representative plots; right panels: quantified MFI. Analysis of control and GRPEL1-depleted PANC-1 (**I**–**K**) and PaTu-8988t (**L**–**N**) cells with or without NAC treatment. **I**, **L** Analysis of cellular ROS by flow cytometry using DCFH-DA staining. **J**, **M** Quantified MFI of cellular ROS. **K**, **N** Immunoblot analysis with the indicated antibodies. Data are presented as means ± SEM for bar graphs from at least three independent experiments. Representative images from three independent biological replicates are shown. ^*^p < 0.05, ^**^p < 0.01, ^***^p < 0.001.

GRPEL1 is required for maintaining mitochondrial respiration [27, 28], which is central to initiating retrograde signaling [29]. We therefore hypothesized that GRPEL1 regulates FASN expression through mitochondrial retrograde signaling. As expected, depleting *GRPEL1* impaired basal respiration, ATP production, and maximal respiration in PANC-1 (Fig. 4A, B) and PaTu-8988t (Fig. 4C, D) cells. In contrast, GRPEL1 overexpression enhanced mitochondrial respiratory function in both PDAC cell lines (Fig. S4A–D). Decreased mitochondrial mass and lower mitochondrial membrane potential were observed in GRPEL1-deficient cells, with the opposite phenotypes evident in GRPEL1-overexpressing cells (Fig. S5A–H). More directly, transmission electron microscopy examination showed that GRPEL1-deficient mitochondria exhibited significant morphological alterations, including swelling, cristae loss, and vacuolization (Fig. S5I, J). Taken together, these findings confirm that GRPEL1 depletion causes mitochondrial damage and dysfunction. Increased reactive oxygen species (ROS) levels, which serve as major retrograde signals due to suppressed mitochondrial respiration [30, 31], were also observed in GRPEL1-depleted PDAC cell lines. Specifically, elevated cellular and mitochondrial ROS levels were found in GRPEL1-depleted PANC-1 (Fig. 4E, F) and PaTu-8988t (Fig. 4G, H) cells. ROS scavenging by NAC treatment effectively lowered ROS levels in PANC-1 cells (Fig. 4I, J) and PaTu-8988t cells (Fig. 4L, M), and restored FASN expression at both the protein (Fig. 4K, N) and mRNA (Fig. S4E, F) levels.

Furthermore, c-Myc depletion increased ROS levels in both PDAC cell lines (Fig. S4G, H). The downregulation of FASN induced by c-Myc depletion was alleviated by NAC supplementation (Fig. S4I–L). Consistently, pharmacological inhibition of c-Myc in PaTu-8988t cells also increased ROS levels (Fig. S4M, N). Moreover, the marked reduction in FASN mRNA levels induced by JQ-1 in PaTu-8988t cells was also rescued by NAC (Fig. S4O). While c-Myc has been established as a direct transcriptional regulator of multiple metabolic enzymes, including FASN [32], its role as a direct transcription factor for FASN in PDAC remains unconfirmed. To address this, we performed a dual-luciferase reporter assay in the same PDAC cell models as used in Fig. 1J, K. The results demonstrate that, at least in our experimental system, c-Myc does not directly regulate FASN transcription (Fig. S4P, Q). ROS accumulation is known to activate signaling pathways that inhibit de novo fatty acid synthesis, such as the AMP-activated protein kinase (AMPK) pathway [33, 34]. In support of this, Liu et al. reported that AMPK activation suppresses FASN expression [35]. We therefore performed KEGG pathway enrichment analysis on relevant GEO datasets, which revealed that genes differentially expressed in response to ROS accumulation were significantly enriched in the AMPK, PI3K-Akt, and MAPK signaling pathways (Fig. S4R, S). Our results establish that GRPEL1, regulated by c-Myc, governs FASN expression through a ROS-mediated retrograde signaling pathway.

### Decreased GRPEL1 levels impaired fatty acid synthesis, leading to inhibited cell proliferation

**Fig. 5:**
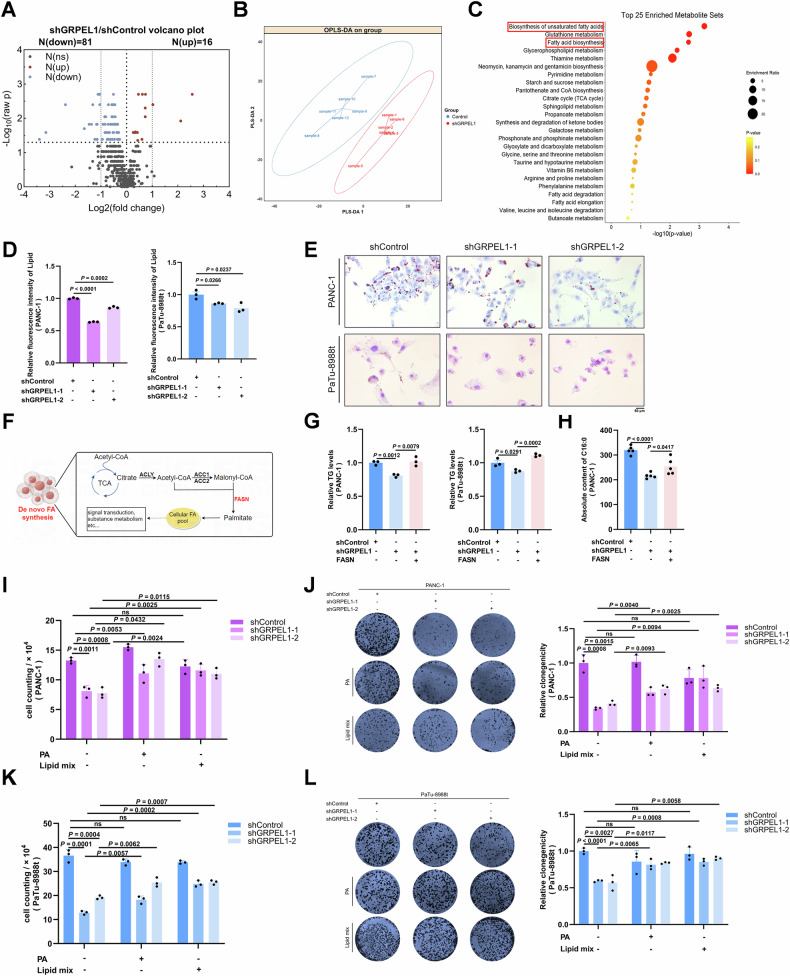
Decreased GRPEL1 levels impaired fatty acid synthesis, leading to inhibited cell proliferation. **A** The metabolite levels in PaTu-8988t cells with or without GRPEL1 depletion were measured by non-targeted metabolomics ( | FC | ≥ 1, *P* < 0.05). **B** Scatter plots of latent variable projections from PLS-DA of untargeted metabolomics data features. **C** A bubble map of KEGG pathway enrichment analysis for the differential metabolites in PaTu-8988t cells upon GRPEL1 depletion. **D** Neutral lipid content measured by flow cytometry in PANC-1 and PaTu-8988t cells upon GRPEL1 depletion, using Bodipy 493/503 staining (data shown as relative MFI). **E** Intracellular lipid droplets in PANC-1 and PaTu-8988t cells with or without GRPEL1 depletion, stained with Oil Red O. **F** A scheme to show the role of FASN in de novo synthesis of fatty acids. **G** Relative triglyceride content in PANC-1 and PaTu-8988t cell models was measured using a commercial kit. **H** C16 levels in PANC-1 cell models were detected by targeted metabolomics. Proliferation (**I**, **K**) and colony formation ability (**J**, **L**) of PANC-1 (upper panels) and PaTu-8988t (lower panels) cells. Cells with or without GRPEL1 depletion were cultured in normal medium, followed by supplementation with exogenous palmitic acid (20 μM) or lipid mixtures (1:500) at the indicated concentrations. Data are presented as means ± SEM for bar graphs from at least three independent experiments. Representative images from three independent biological replicates are shown.

To determine whether GRPEL1 depletion induced FASN-related metabolic alterations, we performed untargeted metabolomics to identify differential metabolites in PaTu-8988T cells with or without GRPEL1 depletion. PDAC cells with GRPEL1 depletion bore a distinct metabolic profile compared to control cells, and a total of 97 differential metabolites were identified (|FC| ≥ 1, *P* < 0.05) (Fig. 5A, B). Furthermore, enrichment analysis of these metabolites revealed that the fatty acid biosynthesis pathway was among the most affected metabolic pathways in GRPEL1-depleted PDAC cells (Fig. 5C). Subsequently, we demonstrated that GRPEL1 deficiency caused impairment of fatty acid synthesis by inhibiting FASN expression through a series of methods, including Bodipy 493/503 staining, oil red O staining, TG content determination, and targeted medium and long chain fatty acid quantification. We observed a reduction in Bodipy 493/503 staining in GRPEL1- depleted PDAC cells (Fig. 5D) and an increase in GRPEL1-overexpressing cells (Fig. S6A). These results support the conclusion that GRPEL1 is required for fatty acid synthesis in PDAC cells. This conclusion was further corroborated by oil red O staining (Fig. 5E) and direct measurement of cellular triglyceride content with or without GRPEL1 knockdown (Fig. S6B, C). Notably, GRPEL1 overexpression partially restored neutral lipid levels in the two PDAC cell lines with c-Myc depletion (Fig. S6D), indicating that the c-Myc/GRPEL1 axis is essential for fatty acid synthesis. Given that FASN is a known master regulator of fatty acid metabolism (Fig. 5F), we then asked whether FASN overexpression could restore lipid and fatty acid levels in PDAC cells with GRPEL1 or c-Myc inhibition. By measuring triglyceride content, we found that FASN overexpression restored lipid levels in PDAC cells with GRPEL1 (Fig. 5G) or c-Myc inhibition (Fig. S6E). Additionally, FASN overexpression partially or fully restored the level of palmitate (PA, C16:0), a direct product of FASN, in GRPEL1-depleted PANC-1 cells (Fig. 5H).

To investigate alterations in lipid components beyond fatty acids, we conducted a comprehensive lipidomic analysis of the PaTu-8988t cell model. Under stringent quality control, a total of 321 lipids were identified, encompassing the following 12 lipid categories: energy storage lipids (e.g., triacylglycerols and free fatty acids), structural/functional membrane lipids (e.g., phosphatidylcholine, phosphatidylethanolamine, sphingomyelin, phosphatidylglycerol, and phosphatidylinositol), and signaling lipids (e.g., ceramide, diacylglycerol, lysophosphatidylcholine, lysophosphatidylethanolamine, and hexosylceramide) (Fig. S7A, B). The results revealed that c-Myc depletion significantly altered the levels of 171 lipids (|Log_2_FC| ≥ 0 and *P* < 0.05), among which 147 were markedly downregulated, including major classes such as triacylglycerols, phosphatidylinositol, lysophosphatidylcholine, and ceramide. Replenishment of GRPEL1 partially restored the levels of these affected lipids (Fig. S7C, E). Under GRPEL1-deficient conditions, 184 lipids were significantly altered (|Log_2_FC| ≥ 0 and *P* < 0.05), with 172 showing notable downregulation across multiple categories, including energy storage lipids, structural/functional membrane lipids, and signaling lipids. The downregulation of free fatty acids was the most pronounced, and this phenotype was rescued by GRPEL1 overexpression, consistent with our earlier findings (Fig. S7D, F). Differential metabolite enrichment analysis further indicated that both c-Myc and GRPEL1 deficiency led to disruptions in multiple lipid metabolic pathways, including glycerophospholipid metabolism and fatty acid synthesis pathways involving linoleic acid, α-linolenic acid, and arachidonic acid (Fig. S7G). Pathway enrichment analysis based on the SMPDB database also confirmed that deficiencies in either c-Myc or GRPEL1 resulted in abnormalities in sphingolipid metabolism, while GRPEL1 deficiency specifically induced alterations in the fatty acid synthesis pathway (Fig. S7H, I).

Moreover, lipid supplementation at least partially restored cell proliferation and colony formation in PANC-1 and PaTu-8988t cells with GRPEL1 depletion (Fig. 5I–L) or c-Myc depletion (Fig. S6F–I). Taken together, these results support our conclusion that fatty acid accumulation underlies the oncogenic activity of GRPEL1 in PDAC cells.

### The GRPEL1/FASN axis regulates fatty acid synthesis, thereby influencing xenograft tumor and PDAC organoid growth

**Fig. 6:**
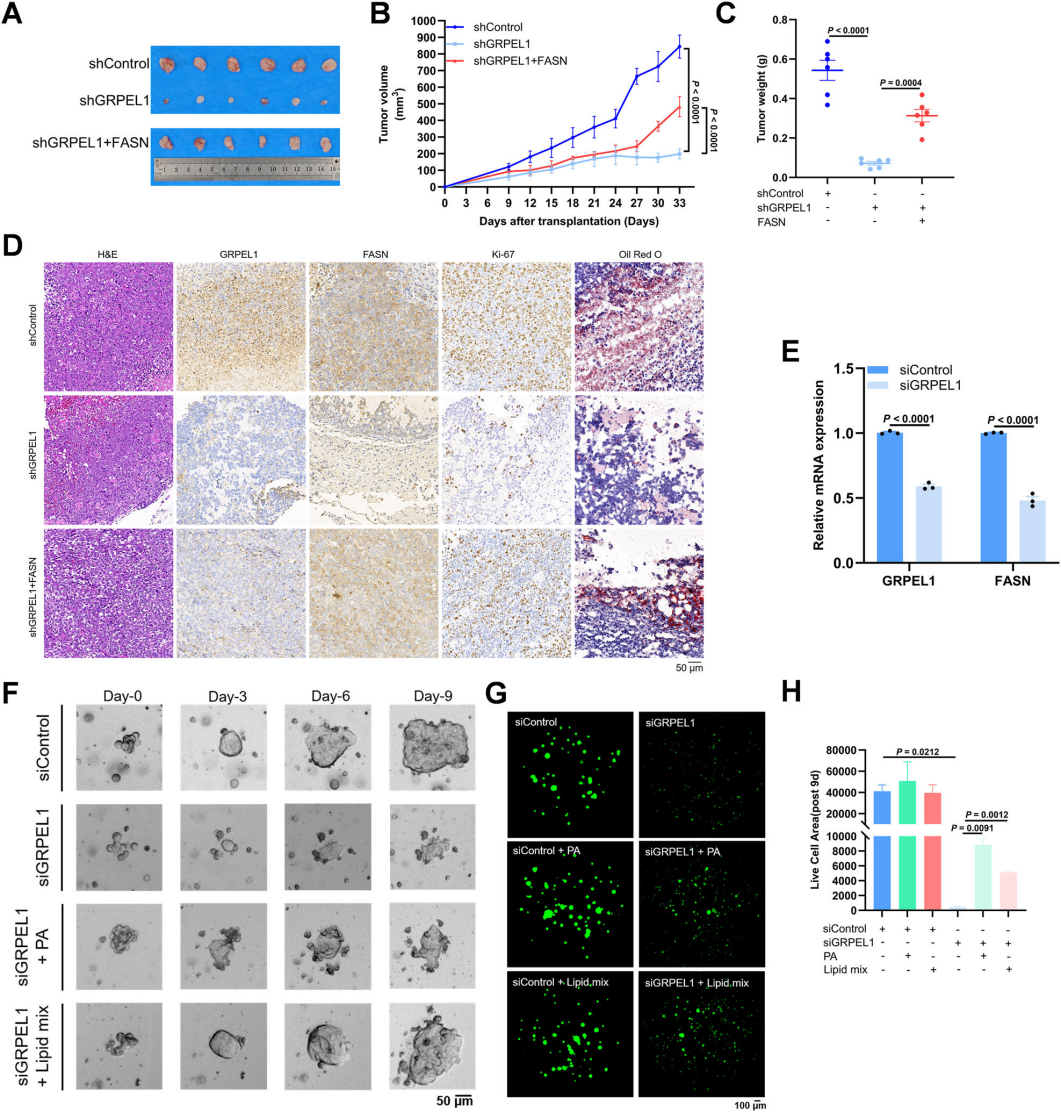
The GRPEL1/FASN axis regulates fatty acid synthesis, thereby influencing xenograft tumor and PDAC organoid growth. The image of PDAC tumors (**A**), tumor volumes (**B**), and tumor weights (**C**) from mice injected with the indicated PaTu-8988t cell models (5 × 10^6^ cells, *N* = 6). **D** Representative images of H&E staining, GRPEL1, FASN and Ki-67 IHC staining, and Oil Red O staining in different groups of xenograft tumor tissues. **E** The expression of *GRPEL1* and *FASN* in PDAC organoids was detected by qPCR. **F** Representative bright-field images show the growth of PDAC organoids with or without GRPEL1 interference and supplemented with PA (50 μM) and lipid mix (1/500) after GRPEL1 interference (*N* = 2). **G**, **H** Viability of control and GRPEL1-knockdown PDAC organoids, treated with or without PA or lipid mix, assessed by Calcein AM/PI staining. Data are presented as means ± SEM for bar graphs from at least three independent experiments. Representative images from three independent biological replicates are shown.

To verify the role of the GRPEL1/FASN axis in promoting PDAC cell growth and fatty acid synthesis in vivo, we subcutaneously implanted PaTu-8988t cells with or without GRPEL1 depletion into NOD/SCID mice. Depleting GRPEL1 or c-Myc inhibited tumor growth, as shown by reduced tumor volume and weight. This inhibition was reversed, at least in part, by either FASN overexpression in GRPEL1-depleted cells or GRPEL1 overexpression in c-Myc-depleted cells (Figs. 6A–C and S8A–C). Consistently, we found that FASN overexpression restored the levels of FASN, cell proliferation, and lipids that were decreased due to GRPEL1 depletion in PDAC tumors (Fig. 6D). Furthermore, overexpression of GRPEL1 partially rescued the decreased GRPEL1 expression, cell proliferation ability, and lipid levels caused by c-Myc depletion (Fig. S8D). We additionally constructed GRPEL1-knockdown cells targeting different sites and, through subcutaneous tumor formation experiments, observed that they also significantly inhibited tumor growth (Fig. S8E–G).

**Fig. 7:**
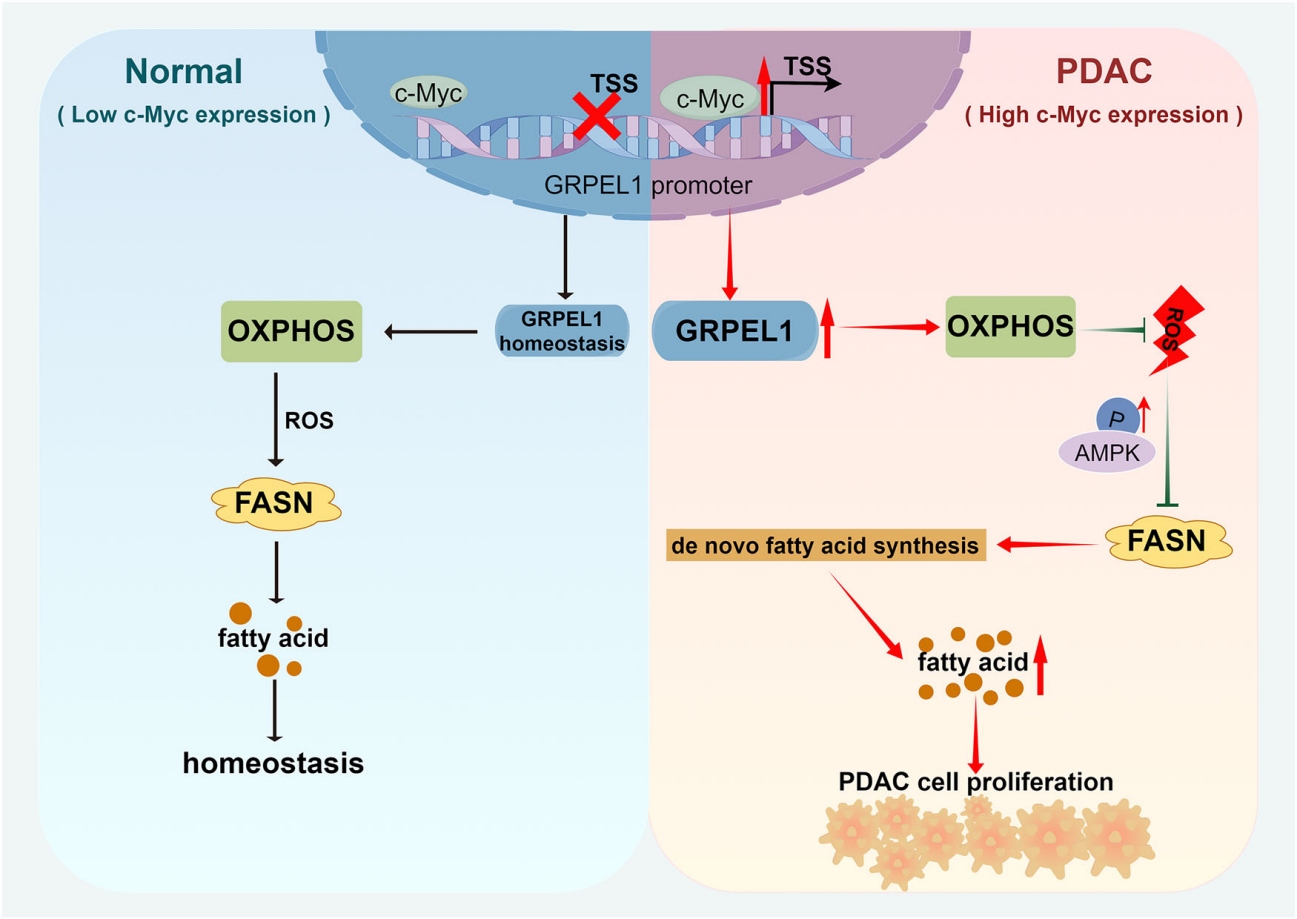
Schematic of the c-Myc/GRPEL1/ROS axis promoting fatty acid synthesis and PDAC growth via FASN expression. Created with FigDraw 2.0 (www.figdraw.com).

Consistently, depleting GRPEL1 in patient-derived PDAC organoids suppressed FASN expression (Figs. 6E and S8H), which again confirmed that GRPEL1 is required for the elevated FASN expression observed in PDAC tumors. Moreover, GRPEL1 depletion inhibited organoid growth, and this inhibition could be rescued by PA or lipid mixture supplementation (Figs. 6F and S8I). Calcein AM/PI staining further confirmed that the viability of GRPEL1-depleted organoids was lower than that of control organoids, which was rescued in part by PA or lipid mixture supplementation (Figs. 6G, H and S8J, K). Together, these results suggest that GRPEL1-regulated fatty acid synthesis, mediated by FASN, promotes PDAC tumor growth in vivo and in patient-derived organoids. Based on a comprehensive analysis of our experimental results in vitro and in vivo, we conclude that c-Myc-driven GRPEL1 overexpression supports PDAC growth by promoting fatty acid anabolism (Fig. 7).

## Discussion

Mitochondria, the central hub of cellular metabolism, play a key role in the metabolic reprogramming of PDAC. Their robust metabolic functions rely on the import of ~1000 to 1300 precursor proteins from the cytosol [36–39]. Dysregulation of mitochondrial protein homeostasis often activates MPQC to restore function, a process critically involved in tumorigenesis and progression [40]. However, several key questions remain: whether MPQC-related genes influence PDAC growth, whether their defects activate or inhibit the MPQC pathway, and whether they regulate metabolism in an MPQC-dependent manner. In this study, we found that GRPEL1, as a MPQC-related protein, drives PDAC growth by promoting FASN-mediated de novo fatty acid synthesis. Mechanistically, c-Myc-regulated GRPEL1 sustains OXPHOS and mitigates ROS accumulation. This, in turn, transcriptionally upregulates FASN expression, thereby promoting de novo fatty acid synthesis.

Dysregulation of the mitochondrial quality control (MQC) represents a pivotal factor in cancer pathogenesis [41, 42]. Classical MQC primarily encompasses three aspects: mitochondrial dynamics, metabolic reprogramming, and MPQC [43]. Under stressful conditions such as hypoxia, nutrient deprivation, oxidative stress, or drug toxicity, mitochondrial protein homeostasis is readily disrupted, frequently triggering the MPQC mechanism to coordinate protein synthesis and degradation. Through a cascade of precise regulatory steps, this process contributes to cellular metabolic remodeling and plays a critical role in the development and progression of various diseases [44–47]. For instance, LONP1 influences the white-to-beige fat transition through the degradation of SDHB (mitochondrial complex II) and subsequent regulation of succinate levels [48]. Mutations in genes such as those encoding the TIM23 complex or DNAJC19, which mediate precursor protein import, impair mitochondrial protein trafficking, leading to energy metabolism disorders and mitochondria-related diseases [49, 50]. Although previous studies have established the role of MPQC-mediated protein homeostasis in metabolic reprogramming and mitochondrial diseases, the specific function of this mechanism in PDAC remains unclear. Our study presents the first systematic investigation into the metabolic regulation of GRPEL1 in PDAC growth. Intriguingly, GRPEL1 deficiency failed to activate the canonical eIF2α/ATF4-mediated MPQC pathway. Instead, it suppressed FASN transcription and translation in a ROS-dependent manner, thereby inhibiting de novo fatty acid synthesis and tumor growth. While we observed clear structural and functional mitochondrial disruption upon GRPEL1 depletion, PDAC cells inherently operate under severe metabolic and secretory pressure and exhibit high basal levels of endoplasmic reticulum (ER) stress [51–53]. Consequently, the mitochondrial stress triggered by GRPEL1 deficiency may be masked by this pre-existing, intense ER stress background, which could explain the lack of significant MPQC activation. Furthermore, the MPQC is a multi-layered, highly redundant regulatory network [13]. The mitochondrial damage induced by GRPEL1 knockdown might not have reached the threshold required to trigger global translational repression or strongly induce key effectors like ATF4. Cells may still adapt to such “subcritical” damage via other compensatory mechanisms.

The K-Ras mutation is a hallmark molecular feature of PDAC, with a mutation frequency exceeding 90% [54]. All PDAC cell lines used in this study carried mutant K-RAS; specifically, PANC-1 harbors the K-RAS G12D mutation and PaTu-8988t the K-RAS G12V mutation. The two organoid models employed were also K-RAS-mutant, consistent with the characteristic molecular background of PDAC. In cancer, a critical collaborator of K-Ras is c-Myc, a multifunctional transcription factor that regulates nearly all aspects of tumor cell metabolism [55]. Multiple studies have established that mutant K-RAS activates c-Myc expression [56–58]. Moreover, c-Myc upregulation has been documented in most PDAC tissues and cells [59, 60]. Consistent with these findings, our tissue microarray analysis also demonstrated enhanced c-Myc expression in PDAC. Based on this established background, we investigated the role of c-Myc in regulating GRPEL1 expression. We discovered, for the first time, that c-Myc functions as a transcription factor for GRPEL1, directly promoting its expression. This finding suggests that c-Myc may participate in MPQC regulation under specific conditions. Furthermore, in contrast to GRPEL1, c-Myc depletion markedly altered the expression of eIF2α and ATF4, implying a direct role for c-Myc in regulating MPQC. This notion is supported by the work of Liu et al., who found that c-Myc overexpression elevated levels of the mtUPR marker HSP60 and activated mtUPR to modulate mitochondrial function [61]. By driving elevated protein synthesis, c-Myc overexpression induces proteotoxic stress, prompting the activation of the parallel PERK-eIF2α and GCN2-eIF2α pathways. These pathways converge on eIF2α phosphorylation, which in turn selectively upregulates ATF4 translation. Thus, c-Myc deficiency undermines this signaling network, diminishing eIF2α phosphorylation and ATF4 output [62]. This finding provides direct evidence that c-Myc is a regulator of the MPQC process.

Notably, the GRPEL1 deficiency reduced both the mRNA and protein levels of FASN, indicating transcriptional regulation of FASN by GRPEL1. Previous work in conditional grpel1 knockout mouse models have observed that transcriptional suppression of the tricarboxylic acid cycle and OXPHOS [29]. Together with the mitochondrial dysfunction observed in GRPEL1-deficient cells, these findings establish a critical role for GRPEL1 in sustaining mitochondrial homeostasis. Impaired OXPHOS commonly leads to ROS accumulation [63], which is consistent with our data. Excessive ROS can subsequently activate signaling pathways such as AMPK. For example, a study reported that inhibiting NDUFA4L2 can induce ROS accumulation in renal clear cell carcinoma [64]; another study showed that atmospheric cold plasma as an inducer of ROS can promote lymphoma cell apoptosis [65]. Pathway enrichment analysis of differentially expressed genes from these two studies revealed that ROS accumulation activates multiple signaling pathways, including AMPK, PI3K-Akt, and MAPK. Phosphorylated AMPK downregulates lipogenic enzymes such as FASN, thus inhibiting lipid accumulation [35, 66]. To directly test whether ROS mediates FASN downregulation upon GRPEL1 deficiency, we treated GRPEL1-deficient cells with the antioxidant NAC. The subsequent reduction in ROS levels led to a recovery of FASN expression. This result confirms that ROS accumulation is the critical mechanism linking GRPEL1 deficiency to the suppression of FASN.

In conclusion, our study demonstrates that GRPEL1 depletion suppresses PDAC growth by impairing fatty acid synthesis. Mechanistically, GRPEL1 governs the expression of a key lipogenic enzyme, FASN, in a ROS-dependent manner, thereby disrupting de novo FA synthesis. Furthermore, we established that GRPEL1 is transcriptionally upregulated by c-Myc in PDAC. Therefore, targeting the c-Myc/GRPEL1/FASN axis represents a promising therapeutic strategy for PDAC.

## Materials and methods

### Cell culture

The human cell lines PaTu-8988t, HEK-293T, and PANC-1 were obtained from the Cell Resource Center of the Chinese Academy of Sciences (Shanghai, China). HEK-293T and PANC-1 cells were maintained in DMEM (Sigma-Aldrich, USA) supplemented with 10% fetal bovine serum (FBS, Sigma-Aldrich). PaTu-8988t cells were cultured in DMEM supplemented with 12% calf serum (CS, Sigma-Aldrich). All media were supplemented with 1% penicillin/streptomycin (Beyotime, Shanghai, China). All cells were cultured at 37 °C in a humidified incubator with 5% CO₂. Cell lines were authenticated using short tandem repeat (STR) profiling, and the absence of mycoplasma contamination was confirmed using the One-Step Mycoplasma Detection Kit (Yeasen, Shanghai, China).

### Vector construction

Short hairpin RNAs (shRNAs) targeting GRPEL1 or c-Myc, along with a non-targeting control shRNA, were synthesized and cloned into the pLKO.1-puro vector by GenScript Biotech (Nanjing, China). The targeting sequences were as follows: shGRPEL1-1, 5′-CGCGTTGAATAGTTCCACATA-3′; shGRPEL1-2, 5′-GATCCAGAAGGTGTTCACAAA-3′; shc-Myc, 5′-CCTGAGACAGATCAGCAACAA-3′. For gene overexpression, the coding sequence of GRPEL1 (Gene ID: 80273) was obtained from GenBank, synthesized, and cloned into the pLVX-IRES-Neo vector (GenScript Biotech). The FASN (Gene ID: 2194) and c-Myc (Gene ID: 4609) overexpression plasmids were obtained from Miaolingbio (Wuhan, China) and Fenghui Biotechnology (Changsha, China), respectively. All plasmid constructs were verified by DNA sequencing prior to use.

### Lentivirus production and infection

HEK-293T cells were used for lentivirus packaging. When the cells reached ~80% confluence in a healthy state, they were seeded into six-well plates. After the cell density exceeded 60% confluence, lentivirus packaging was performed. We used Lipofectamine™ 3000 (Thermo Fisher Scientific, USA) to co-transfect the target plasmid with two packaging plasmids (psPAX2 and pMD2.G, Addgene, USA) at a ratio of 4:3:1 into the HEK-293T cells, with a total plasmid amount of 2.5 μg. The supernatants were collected at 24 and 48 h post transfection and filtered through a 0.45 μm filter to obtain the lentiviral particles. The lentiviral titer was determined using a titer assay kit (Biodragon, Beijing, China). Well-growing PDAC cell lines were then seeded in 24-well plates at a density of ~1 × 10⁵ cells per well. The volume of the viral solution was calculated based on the multiplicity of infection for each cell line. The lentivirus was added to the cells, which were starved for 12 h before the medium was replaced with a complete medium. After 24 h, the medium was replaced with fresh complete medium, and the cells were cultured for a total of 72 h. Successfully infected cells could be selected using puromycin, G418, or by monitoring green fluorescent protein expression.

### siRNA transient transfection

Lipofectamine™ RNAiMAX (Thermo Fisher Scientific) was used for siRNA transient transfection. Specifically, Opti-MEM mixed with Lipofectamine™ RNAiMAX was designated as mixture 1, and siRNA diluted in Opti-MEM was designated as mixture 2. After incubation for 5 min, the two mixtures were combined and incubated for an additional 15 min to allow complex formation, before being added to the cells.

### Western blotting

The cell suspension was centrifuged to remove the medium, and the cell pellets were collected. The pellets were resuspended in RIPA lysis buffer (Beyotime) and incubated on ice for 20 min. Subsequently, the lysates were centrifuged again to collect the supernatant containing the total protein. Protein concentration was quantified using the BCA^TM^ Protein Assay Kit (Thermo Fisher Scientific). Protein samples (2 μg/μL) were separated by sodium dodecyl sulfate-polyacrylamide gel electrophoresis (SDS-PAGE) and transferred onto PVDF membranes (Merck Millipore) for further analysis. To block nonspecific binding, the membranes were incubated in a 5% skim milk solution. The primary and secondary antibodies were diluted to their optimal working concentrations. The membranes were first incubated with the primary antibody at 4 °C overnight. After washing the next day, the membranes were incubated with the secondary antibody at room temperature for the specified duration before imaging. Finally, the protein bands were visualized using a gel imager (Saizhi, Beijing, China). The primary antibodies, including anti-c-Myc (67447-1-lg), anti-GRPEL1 (12720-1-AP), and anti-FASN (10624-2-AP), were purchased from Wuhan Sanying Biotechnology (ProteinTech, Wuhan, China). The anti-GAPDH antibody (ab9484) was obtained from Abcam (USA). The secondary antibodies, goat anti-rabbit IgG (A0208) and goat anti-mouse IgG (A0216), were provided by Beyotime. The grayscale values of the protein bands were determined using ImageJ software. All Western blot experiments were independently repeated at least three times, and a representative result is shown in the figure.

### Total RNA extraction and RT-qPCR

Total RNA was extracted using Trizol (Takara, Japan) and subsequently reverse-transcribed into cDNA using the PrimeScript™ IV 1st Strand cDNA Synthesis Mix Kit (Takara). RT-qPCR was performed on a cobas z480 system (Roche, Germany) with ChamQ Universal SYBR qPCR Master Mix (Vazyme, Nanjing, China). The corresponding primer sequences are listed in the Supplementary Material.

### Cell counting

Cells were plated in 12-well dishes at a density of 1 × 10⁴ cells per well, with three replicates per group. After the cells adhered to the wall, the first cell count was conducted. Subsequently, cells were counted every 24 h for a total of five times. For each counting, 100 μL of trypsin was added to each well to digest the cells, and the digestion was terminated by adding 400 μL of medium containing FBS or CS. Then, 500 μL of the cell suspension was collected, and 10 μL of it was loaded onto a Neubauer counting plate (Marienfeld, Germany) for cell counting under a microscope. Unless otherwise specified, the cell number at 72 h was uniformly used for statistical analysis.

### Colony formation assays

Healthy, actively growing cells were seeded in 6-well plates at a density of 1 × 10³ cells per well and incubated for 2 weeks until visible colonies had formed. The culture medium was carefully removed, and the cells were gently rinsed with PBS and fixed with 4% paraformaldehyde. The colonies were then stained with 0.1% crystal violet (Solarbio, Beijing, China). After staining, the dye solution was discarded, and the samples were thoroughly washed with distilled water. The cell colonies were quantitatively analyzed using ImageJ software.

### Cell cycle analysis

Cells in the logarithmic growth phase were harvested for cell cycle analysis. Briefly, 500 μL of cell suspension was fixed by adding 1.5 mL of pre-cooled absolute ethanol drop by drop, with gentle mixing, followed by incubation at 4 °C overnight. The fixed cells were then stained using a cell cycle detection kit (Beyotime) according to the manufacturer’s instructions and analyzed by flow cytometry. Data were analyzed using FlowJo software.

### Cell apoptosis assay

The apoptotic status of cells was detected using an apoptosis staining kit (Dojindo, Japan). Before detection, cells were collected by centrifugation at a low speed (300–500 × *g*) and washed at least three times with pre-cooled PBS. The cell pellet was then resuspended in 500 μL of Annexin V Binding Solution. A 100 μL aliquot of the cell suspension was incubated with 5 μL of Annexin V-FITC conjugate and 5 μL of PI solution in the dark for 15 min. Following incubation, 400 μL of Annexin V Binding Solution was added. The samples were then analyzed by flow cytometry, and the apoptosis rate was determined using FlowJo software.

### Seahorse based extracellular flux analysis

The oxygen consumption rate (OCR) of cells from the control and experimental groups was measured using a Seahorse XFe96 extracellular flux analyzer (Agilent, USA). Specifically, the Cell Mito Stress Test Kit (Agilent) was employed for the assay. Cells were seeded into XF 96-well culture plates at a density of 4 × 10⁴ cells per well and cultured overnight. OCR measurements were performed the following day after the application of different drugs. The final concentrations of the modulators used in the assay were as follows: 1.0 μM FCCP, 1.0 μM oligomycin, and 0.5 μM of both rotenone and antimycin A. For each experimental condition, at least three replicate wells were set up, and the entire experiment was independently repeated at least three times. The resulting data were analyzed using the Seahorse WAVE software.

### Determination of ROS production

ROS levels were assessed by staining cells with DCFH-DA (Beyotime) for total ROS and MitoSOX™ Red (Thermo Fisher Scientific) for mitochondrial ROS, respectively. Briefly, 10 μM DCFH-DA and 5 μM MitoSOX™ Red working solutions were prepared. Cells were resuspended in the respective working solutions and incubated at 37 °C in the dark: for 15 min with DCFH-DA and for 30 min with MitoSOX™ Red. After incubation, the cells were washed and analyzed by flow cytometry. The data were analyzed using FlowJo software.

### Mitochondrial biogenesis assay

To quantify mitochondrial mass, cells in the logarithmic growth phase were stained with 100 nM MitoTracker Deep Red FM (Thermo Fisher Scientific) at 37 °C for 30 min and subsequently subjected to flow cytometric analysis. Data from 10,000 cellular events were collected, and the median fluorescence intensity was calculated using FlowJo software.

### Analysis of mitochondrial membrane potential

The mitochondrial membrane potential was assessed using JC-1 staining (Beyotime) according to the manufacturer’s instructions. Briefly, cells were resuspended in the 1× JC-1 working solution at a density of 1 × 10⁵ to 5 × 10⁵ cells per sample and incubated at 37 °C in the dark for 20 min. After incubation, the cells were washed and analyzed by flow cytometry. Data analysis was performed using FlowJo software.

### Transmission electron microscopy (TEM)

The samples were fixed overnight at 4 °C in 2.5% glutaraldehyde. They were then washed several times with phosphate buffer and post-fixed in 1% osmium tetroxide for 1–2 h. After subsequent rinses with phosphate buffer, the samples were dehydrated through a graded ethanol series, followed by treatment with absolute ethanol and anhydrous acetone. Finally, the specimens underwent sequential processing, including infiltration, embedding, ultrathin sectioning, staining, and observation under a transmission electron microscope for image acquisition.

### CHIP and CHIP-qPCR

ChIP experiments were performed using the SimpleChIP® Plus Enzymatic Chromatin IP Kit (Cell Signaling Technology, USA). Briefly, when cell density in the large dish reached ~80%, the original medium was discarded. Cells were fixed with 1% paraformaldehyde (prepared by dilution in fresh medium) and the cross-linking was quenched by adding 10× glycine. The cells were then washed with 2 mL of PBS containing 10 μL of 200× PIC and collected by centrifugation at 2000 × *g* for 5 min at 4 °C. The cell pellet was resuspended in 1 mL of 1× Buffer A (supplemented with 0.5 μL of 1 M DTT and 5 μL of 200× PIC), incubated on ice for 10 min, and then centrifuged again under the same conditions to isolate the nuclei. The nuclei were resuspended in 1 mL of 1× Buffer B (with DTT), centrifuged, and the resulting pellet was resuspended in 100 μL of 1× ChIP Buffer. DNA was digested with micrococcal nuclease, and the digestion was terminated by adding 0.05 M EDTA. The chromatin was fragmented by sonication. The samples were incubated with anti-IgG or anti-c-Myc antibodies at 4 °C for 14–16 h. The next day, 30 μL of agarose beads were added to each reaction mixture for co-immunoprecipitation. After a series of washes with high-salt and low-salt buffers, the cross-links were reversed, and the chromatin was purified. Finally, qPCR analysis was performed on the DNA fragment corresponding to the c-Myc binding motif (-988 to -977 bp, GGCCACGCGGT) in the GRPEL1 promoter. The detailed primer sequences are provided in the Supplementary Material.

### Dual luciferase reporter assay

The transcriptional activity of *c-Myc* was assessed using a dual-luciferase reporter assay system [67]. The 2 × 10^3 ^bp DNA fragment containing the potential *c-Myc* binding motif within the *GRPEL1* promoter was synthesized by Fenghui Biotech and cloned into the pGL4.20 vector (Promega, USA). PaTu-8988t cells were co-transfected with the *GRPEL1* promoter-pGL4.20 construct, the pRL-TK vector, and either *c-Myc* siRNA or a *c-Myc* overexpression vector. After 24 h, the cells were collected by centrifugation and lysed with 250 μL of Passive Lysis Buffer. Then, 20 μL of the lysate was transferred to a well of a 96-well plate, and the background luminescence was measured using a microplate reader. Subsequently, 100 μL of Luciferase Assay Reagent II was injected into the well, and the firefly luminescence (Lum1) was measured immediately after brief mixing. Next, 100 μL of Stop & Glo Reagent was added to the same well, and the Renilla luminescence (Lum2) was measured. The final reporter activity was calculated as the radio of firely to Renilla luminescence (Lum1/Lum2), normalized to the total protein concentration determined by a bicinchoninic acid (BCA) assay.

### PDAC tissues microarray and immunohistochemical (IHC) analysis

The ethically approved human PDAC tissue microarray (TMA) was obtained from Shanghai Kaiwang Biotech (ethical approval number: KW2024126) and contained 67 paired samples of PDAC tissues and corresponding adjacent pancreatic tissues. Paraffin-embedded tissue sections, ~3–6 μm in thickness, were prepared for IHC staining. The sections were incubated overnight with anti-c-Myc, anti-GRPEL1, and anti-FASN antibodies (ProteinTech), followed by a 1 h incubation with secondary antibodies. After washing with PBS, the sections were stained with diaminobenzidine (DAB, Beyotime). The stained sections were then sequentially dehydrated through a graded ethanol series, cleared in xylene, and mounted with neutral resin. Finally, all sections were scanned using a Pannoramic MIDI scanner (3DHISTECH, Hungary) and imaged with the CaseViewer software. Patient information is provided in the Supplementary Material.

### Quantitative proteomics analysis

We outsourced the 4D label-free quantitative proteomic analysis to Applied Protein Technology, Inc. (APTBIO, Shanghai, China). The SDT lysate containing 4% SDS was neutralized to pH 7.6. Cells were then suspended in this solution and lysed on ice for 10 min before the protein concentration was measured. The extracted proteins were separated by SDS-PAGE and stained with Coomassie Brilliant Blue R-250 (Beyotime) for visualization. Proteins from each sample were digested enzymatically following the Filter-Aided Proteome Preparation (FASP) protocol [68]. The resulting peptide mixture was desalted using a C18 cartridge, freeze-dried, and redissolved in 0.1% formic acid. The peptide concentration was estimated by measuring the absorbance at 280 nm. For liquid chromatography separation, an HPLC system was employed. The mobile phase consisted of two solutions: Solution A (0.1% formic acid) and Solution B (0.1% formic acid in 99.9% acetonitrile). The chromatographic column was equilibrated with Solution A. Separation was performed using a C18 reversed-phase analytical column (EASY column; 25 cm length, 75 μm inner diameter, 1.9 μm particle size; Thermo Fisher Scientific) at a flow rate of 300 nL/min. Separated samples were analyzed by a timsTOF Pro mass spectrometer (MS). The detection mode was set to positive ion. The instrument settings were configured with the following specifications: the ion source voltage was set to 1.5 kV, and the MS scan range was set from 100 to 1700 *m*/*z*. Data were acquired in Parallel Cumulative Serial Fragmentation (PASEF) mode. Key acquisition parameters included an ion mobility range (1/K0) of 0.6–1.6 Vs/cm², with each full MS scan triggering the collection of 10 subsequent MS/MS spectra. The dynamic exclusion time was set to 24 s. MaxQuant was used to process and analyze the raw proteomic data. Subsequently, the data were subjected to bioinformatic analysis.

### Untargeted metabolomics analysis

The CalOmics platform (Calibra, Hangzhou, China) was used for untargeted metabolomic quantification. Cell pellets were collected by centrifugation and homogenized by sonication. A threefold volume of methanol was added to the samples and mixed thoroughly, followed by centrifugation. A 100 μL aliquot of the supernatant was transferred to each well of a plate. The samples were then dried under a nitrogen stream and redissolved. The resulting samples were analyzed by UPLC-MS/MS. Metabolites were detected using an ACQUITY 2D UPLC (Waters, USA) coupled to a Q Exactive (QE) hybrid Quadrupole-Orbitrap mass spectrometer (Thermo Fisher Scientific). After the analysis, the data were pre-processed. Following successful quality control, the raw data were processed and normalized using the proprietary platform. The quantitative metabolomic data were analyzed by statistical and bioinformatic methods. R (version 3.4.1) was used for statistical analysis and visualization of all data. Data visualization included volcano plots, PCA scatter plots, and PLS-DA/OPLS-DA scatter plots. Pathway enrichment analysis of differential metabolites was performed based on the KEGG database, using the MetPA or Pathview packages [69, 70].

### Targeted fatty acid content determinations

Medium and long chain FAs were determined by Calibra Lab on its self-developed targeted metabolomics platform. Cells were prepared in at least five replicates to ensure the reliability of the results. Sample preparation was performed according to Calibra’s protocol. Specifically, samples after flash-freezing in liquid nitrogen were mixed with methanol, methyl tert-butyl ether, and 36% phosphoric acid/water, and the supernatants were collected by centrifugation in a precooled centrifuge. Samples were dried under a nitrogen stream, vortexed after the addition of a 15% boron trifluoride in methanol solution, and then heated in an oven at 60 °C for half an hour. After the samples were cooled to room temperature (RT), hexane and a saturated salt solution were added. The mixture was vortexed again and centrifuged, and the upper organic phase was taken for GC-MS analysis. An Agilent 7890B gas chromatography system (Agilent) coupled to an Agilent 5977A mass spectrometer (Agilent) was used for the separation and analysis of the targeted metabolites. QuantAnalysis software was used to process the raw data and calculate the targeted metabolite content based on the standard curve. The final results were normalized to the cell number.

### RNA sequencing

Total RNA was extracted from negative control and GRPEL1-depleted PaTu-8988t cells. Sequencing was performed by Beijing Novogene Biotechnology Co., Ltd (Beijing, China). Specifically, the Agilent 2100 bioanalyzer (Agilent) was used to detect the integrity and total amount of the RNA. Then a series of subsequent steps were performed, including library preparation for transcriptome sequencing, Illumina sequencing, data quality control, sequence alignment to the reference genome, and gene expression level quantification. Subsequently, the sequencing data were subjected to bioinformatics analysis according to the experimental requirements.

### Gene set enrichment analysis (GSEA)

GSEA was performed using GSEA software version 4.4. This method evaluates the expression levels of specific gene sets between control and GRPEL1-depleted cells. The normalized enrichment scores (NES) were generated by performing 1000 gene set permutations. Typically, a significant GSEA result was defined by a *P*-value < 0.05 and |NES| > 1.

### Oil red O staining

Once cells in the 12-well plate reached ~80% confluency, the culture medium was removed, and the wells were rinsed three times with PBS. The cells were then fixed with 4% paraformaldehyde for 15 min. Lipid droplets were stained using an Oil Red O staining kit (Solarbio). Specifically, an Oil Red O working solution was prepared by diluting the stock solution to 60% with distilled water, followed by filtration. Cells were treated with 60% isopropanol for 2–5 min before the solution was removed. An adequate amount of the Oil Red O working solution was then added to cover the cells, which were incubated in the dark for at least 30 min at room temperature. After the stain was discarded, the cells were rinsed with PBS. Finally, the nuclei were counterstained with hematoxylin. The same staining procedure was applied to frozen sections.

### Preparation of PA

PA (MCE, USA) was prepared according to a protocol previously described by Cousin et al. [71]. Briefly, 0.1 M sodium hydroxide was prepared first. Then, 100 mM PA was dissolved in the sodium hydroxide solution and heated at 70 °C to facilitate dissolution. A 10% (weight/volume) fatty acid-free bovine serum albumin (BSA, Sigma-Aldrich) solution was prepared and solubilized by heating at 55 °C. The final concentration of PA was adjusted by mixing with the 10% fatty acid-free BSA solution.

### Determination of TG content

Intracellular triglyceride content was measured using the EnzyChrom™ Triglyceride Assay Kit (BioAssay Systems, USA). Cell pellets were lysed with RIPA buffer, and the supernatant was collected. The reaction system was prepared according to the manufacturer’s protocol, and the absorbance at 570 nm was recorded. The final data were normalized using the BCA method.

### Bodipy 493/503 Staining

The content of neutral lipids was measured using the lipophilic fluorescent dye BODIPY 493/503 (Molecular Probes, USA). Cell pellets were resuspended in a 2 μM BODIPY working solution and incubated at 37 °C in the dark for 30 min. The mean fluorescence intensity (MFI) was determined by flow cytometry. The data were analyzed using FlowJo software.

### Lipidomics

For lipidomics analysis, each cell sample was prepared with a minimum of five independent replicates. Specifically, 5 million cells were centrifuged to obtain the cell pellet, which was then washed three times with pre-chilled PBS and rapidly frozen in liquid nitrogen. The samples were subsequently sent to Metabo-Profile Biotechnology Co., Ltd (Shanghai, China) for lipidomics quantification. Data processing and statistical analysis were performed using the company’s in-house developed software, iMAP (version 1.0, Metabo-Profile, Shanghai, China).

### Construction and culture of tumor organoids

The PDOs used in this study were obtained from Xellar Biosystems (Shanghai, China). All experimental procedures were reviewed and approved by the relevant Ethics Committee (ethical approval number: 伦审2020科060). Specifically, tumor organoids were constructed as previously described [72]. Fresh pancreatic cancer surgical specimens were stored in tissue preservation solution and processed within 8 h. The surgical samples were washed three to four times with tissue cleaning solution to remove necrotic tissue, calcifications, and blood clots. The tissues were minced into ~1 mm³ pieces. A digestion enzyme solution, with a volume five times that of the tissue, was added, and the mixture was incubated at 37 °C for 30 min with shaking. The tubes were briefly vortexed at 5-min intervals during the incubation. The digestion mixture was filtered through a 100 μm cell strainer, and the digestion process was terminated by adding an equal volume of tissue cleaning solution. The cell pellets were collected by centrifugation. If red blood cells were present, 1–2 mL of red blood cell lysis buffer (Biosharp, Hefei, China) was added and incubated on ice for 2 min. The lysis process was terminated by adding twice the volume of DPBS (Servicebio, Wuhan, China), and the cell pellets were collected by centrifugation again. Based on the cell counting results, an appropriate number of cells were taken and mixed with Matrigel (Corning, USA). The cell-Matrigel mixture was then plated, and complete medium was added for culture. Most organoids were passaged or frozen when they reached ~200 μm in size or after 5–7 days of culture.

### siRNA transfection and proliferation assessment in organoids

siRNA transfection: The organoids were digested into single cells or small cell clusters, collected by centrifugation, and aliquoted at a density of 5000 cells per well (for a 96-well plate). An appropriate amount of siRNA was diluted in Opti-MEM. Simultaneously, Lipofectamine 2000 (Thermo Fisher Scientific) was mixed with Opti-MEM in a separate tube and incubated at room temperature for 5 min. The two solutions were then combined to form the transfection complex. The organoid cells were incubated with this complex at 37 °C for 4–6 h. Following incubation, the cell pellets were collected by centrifugation, resuspended in Matrigel, and seeded into a 96-well plate.

Proliferation assessment: According to the experimental design, organoids in the drug treatment groups were exposed to 50 μM PA overnight, after which the medium was replaced with complete medium. Alternatively, a lipid mixture diluted 500-fold in complete medium was added. Organoids treated with complete medium alone served as the negative control. Bright-field images were acquired using an EVOS M7000 microscope on days 0, 3, 6, and 9 post-treatment. On day 9, the organoids were also stained with Calcein-AM, PI, and Hoechst 33342 for fluorescence imaging. The fluorescence images were analyzed using the Xellar Biosystems image analysis platform (Xellar Biosystems, Shanghai, China). The viable cell area at the experimental endpoint was quantified to compare the proliferative capacity of the tumor organoids across the different groups.

### Animal experiments

PaTu-8988t cells, confirmed to be free of mycoplasma contamination, were seeded in large dishes. When the cells reached ~80% confluency, they were collected, washed with pre-cooled PBS, and resuspended in a 1:1 mixture of pre-cooled PBS and Matrigel (Corning, USA). The cell density was adjusted to 5 × 10⁶ cells per 200 μL. A 200 μL aliquot of the cell suspension was then injected subcutaneously into the shoulder region of ~4-week-old mice. One to two weeks post-injection, slightly raised tumors became visible under the skin. Tumor size was measured every 3 days. The tumor volume was calculated according to the following formula: Volume = (Length × Width²) × 0.5236. When the tumor volume reached ~1000 mm³, the mice were euthanized. The tumors were dissected, photographed, and weighed for subsequent experiments. This in vivo study was approved by the Ethics Committee of Wenzhou Medical University (wydw2023-0106).

### Statistical analysis

The results are expressed as the mean ± standard error of the mean (SEM), and each experiment was replicated at least three times under independent conditions. The sample size (*N*) represents the number of independent biological replicates. For in vitro studies, this refers to organoids derived from PDAC patients; for in vivo studies, it refers to the mice used in the animal experiments. For comparisons involving multiple groups, statistical significance was assessed using one-way analysis of variance (ANOVA), while differences between two groups were evaluated with a two-tailed Student’s *t*-test. All analyzes and graphical representations were generated using GraphPad Prism (version 10.1.2; GraphPad Software, USA). A *p*-value of less than 0.05 was considered statistically significant.

## Supplementary information

Supplementary information

Supplementary Materials

westblot original figure

## Data Availability

The data that support the findings of this study are available from the corresponding author upon reasonable request.
