# Supplementary material for: c-Myc/GRPEL1 maintains fatty acid synthesis via FASN to support PDAC cell proliferation
Source: Cell Death Dis. 2026 Feb 5;17(1):205. doi: 10.1038/s41419-026-08439-0 (PMC12894993; doi:10.1038/s41419-026-08439-0)
Supplement: Supplementary file 1 — Supplementary information [file 41419_2026_8439_MOESM1_ESM.docx]

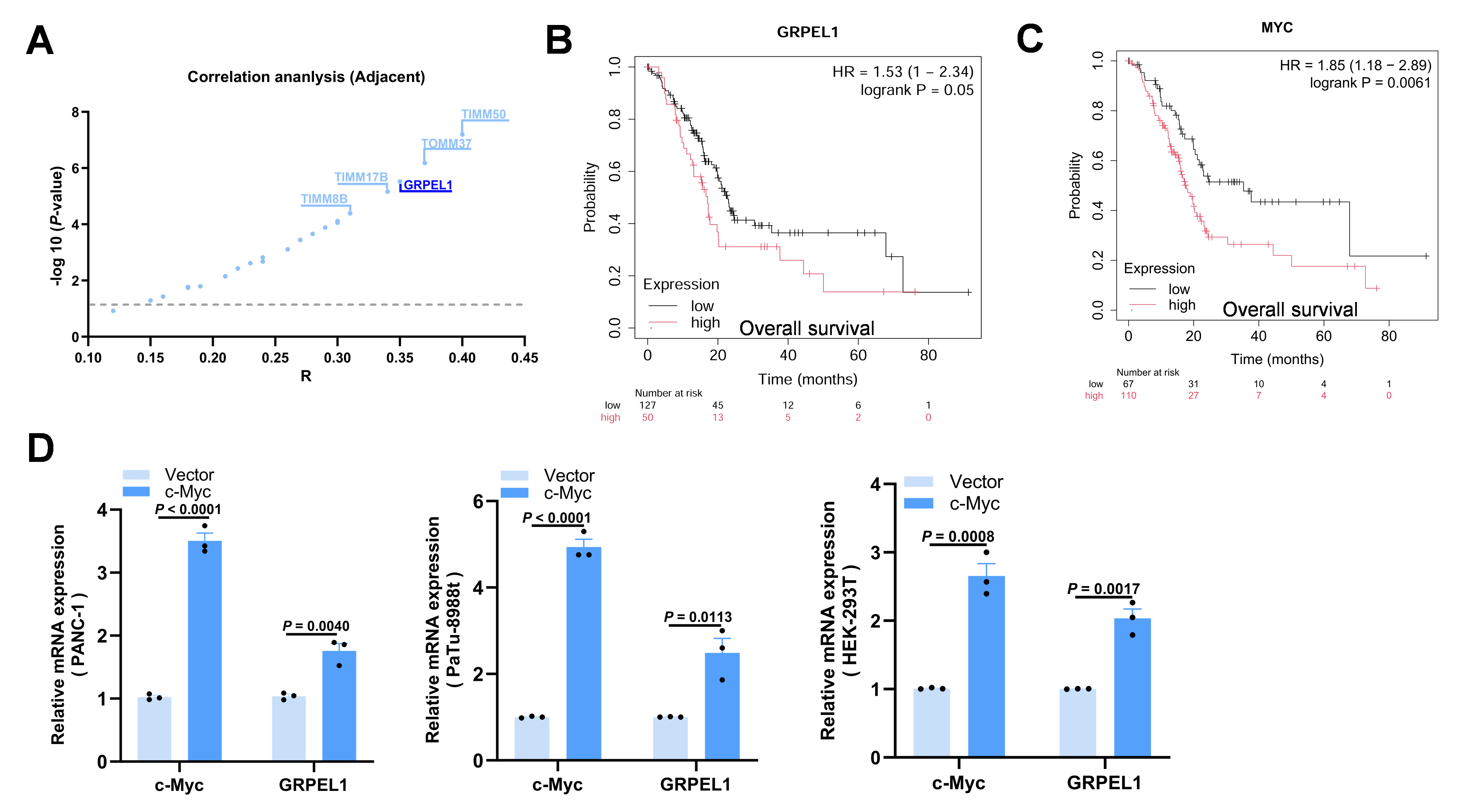


**Supplementary Figure 1.**

(A) Correlation analysis of c-Myc and 23 DEGs in adjacent tissues (GTEx, n=171).

(B, C) Kaplan-Meier survival analysis of PDAC patients based on high or low expression of GRPEL1 (B) and c-Myc (C), analyzed using the Kaplan–Meier plotter database.

(D) qPCR analysis of *GRPEL1* mRNA levels in PANC-1, PaTu-8988t, and HEK-293T cells with or without c-Myc overexpression.

Data are presented as means ± SEM for bar graphs from at least three independent experiments.


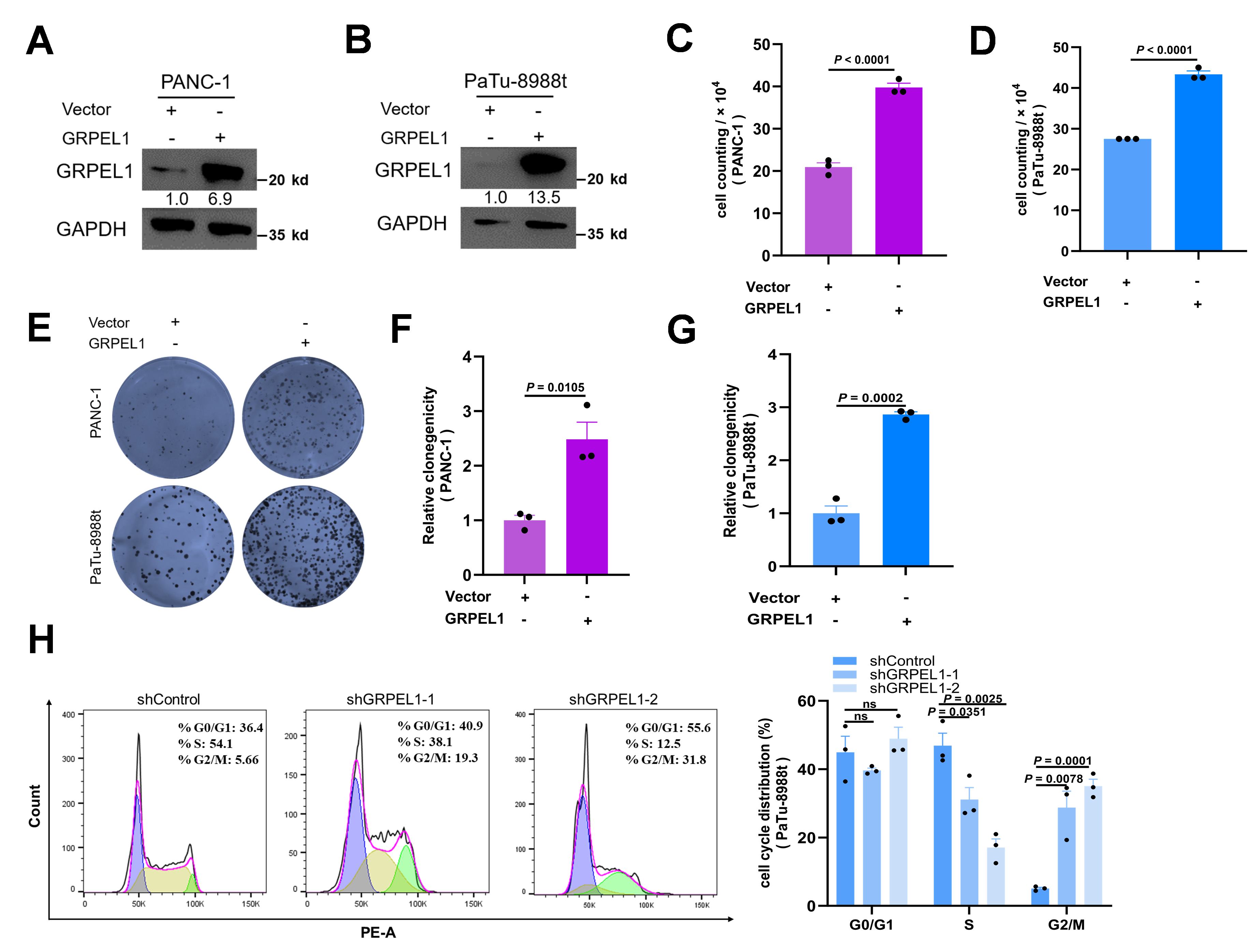


**Supplementary Figure 2.**

(A, B) Immunoblot analysis of GRPEL1 expression with or without GRPEL1 overexpression in PANC-1 and PaTu-8988t cells. GAPDH was used as an internal control.

(C, D) Cell proliferation of PANC-1 and PaTu-8988t cells with or without GRPEL1 overexpression.

(E) Colony formation assay assessing the long-term growth of PANC-1 and PaTu-8988t cells with or without GRPEL1 overexpression.

(F, G) Quantification of the colony number.

(H) Cell cycle analysis of PaTu-8988t cells with or without GRPEL1 depletion, determined by flow cytometry.

Data are presented as means ± SEM for bar graphs from at least three independent experiments. Representative images from three independent biological replicates are shown.


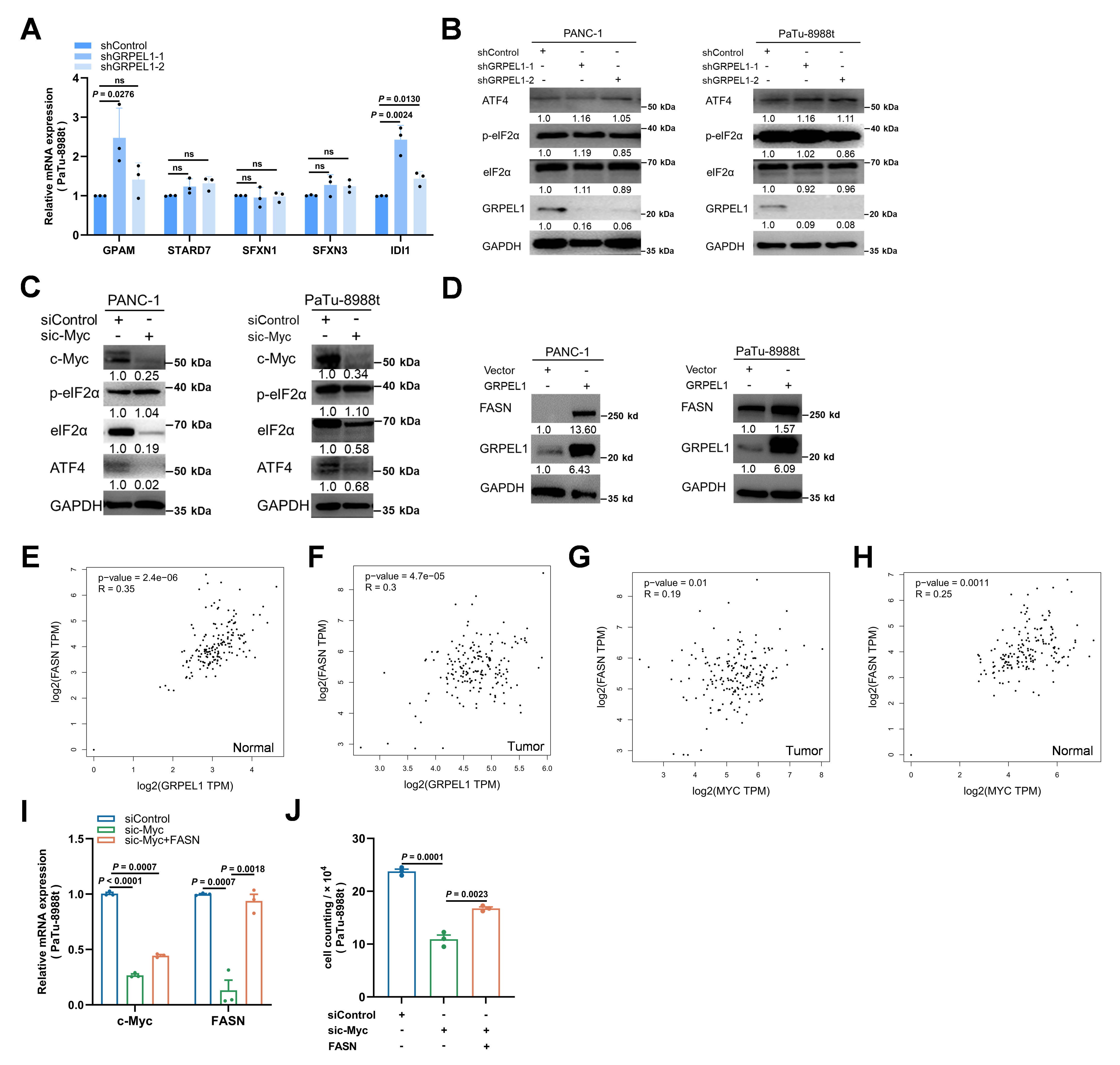


**Supplementary Figure 3.**

(A) qPCR analysis of the relative mRNA levels of GPAM, STARD7, SFXN1, SFXN3, and IDI1 in PaTu-8988t cells upon GRPEL1 depletion.

(B-D) Immunoblot analysis of PANC-1 and PaTu-8988t cells using the indicated antibodies.

(E-H) Correlation analysis between GRPEL1 and FASN (E, F) or c-Myc and FASN (G, H) in peri-tumoral pancreatic tissues and PDAC tissues, performed using the GEPIA 2.0 database.

(I) qPCR analysis of the relative mRNA levels of c-Myc and FASN in the indicated PaTu-8988t cell models.

(J) Proliferation of the indicated PaTu-8988t cell models.

Data are presented as means ± SEM for bar graphs from at least three independent experiments. Representative images from three independent biological replicates are shown.


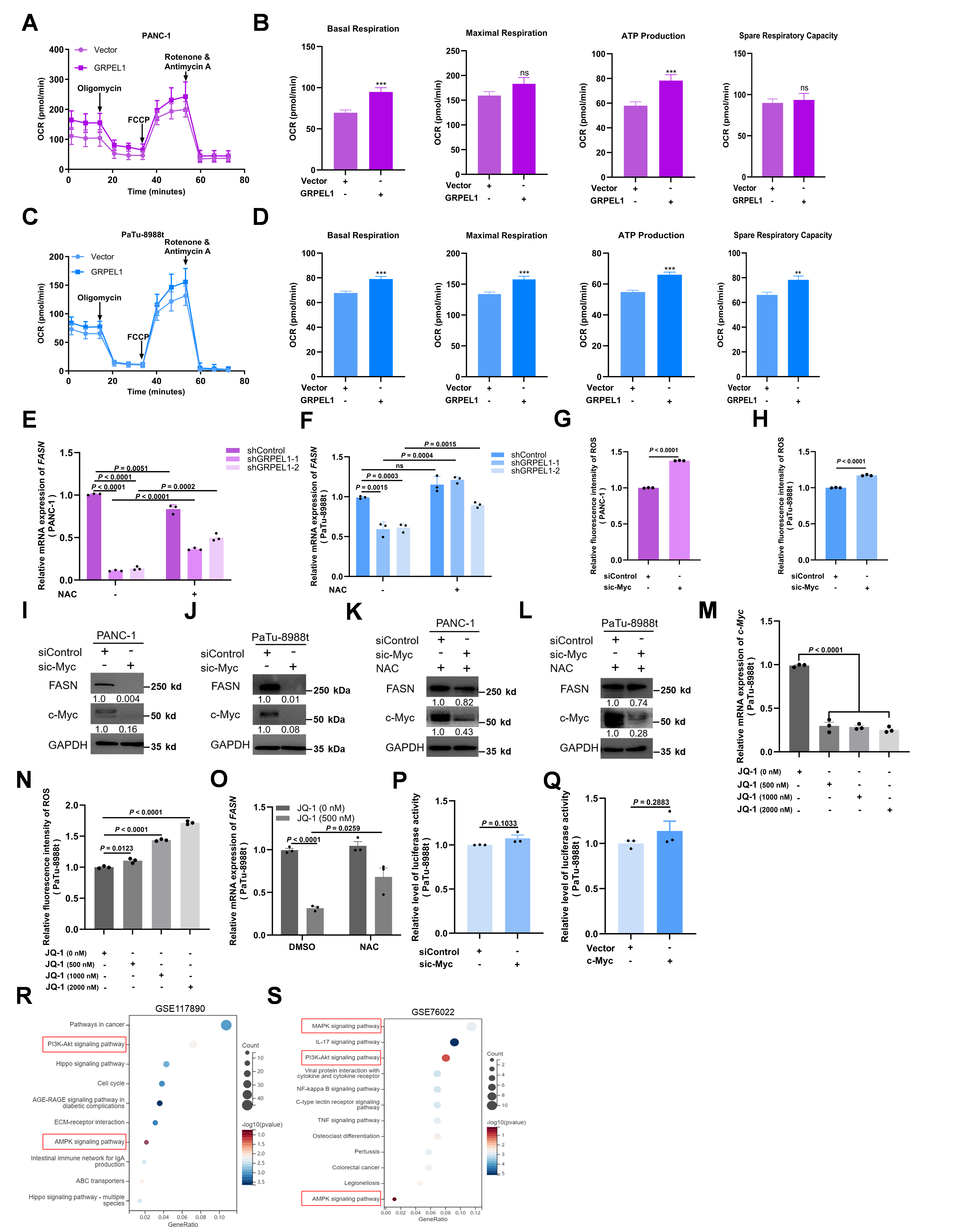


**Supplementary Figure 4.**

(A-D) Seahorse analysis of OCR in PANC-1 (A, B) and PaTu-8988t (C, D) cells upon GRPEL1 overexpression. (A, C) OCR profiles. (B, D) Quantification of basal respiration, maximal respiration, ATP production, and spare respiratory capacity.

(E, F) Flow cytometric analysis of cellular ROS levels in PANC-1 and PaTu-8988t cells using DCFH-DA staining.

(G, H) Flow cytometric analysis of cellular ROS formation through DCFH-DA staining in PANC-1 and PaTu-8988t cells with or without *c-Myc* interference.

(I-L) Immunoblot analysis of control and c-Myc-depleted PANC-1 and PaTu-8988t cells treated with or without NAC, using the indicated antibodies.

(M) qPCR analysis of *c-Myc* mRNA levels in PaTu-8988t cells treated for 72h with the indicated concentrations of JQ-1.

(N) Cellular ROS levels in PaTu-8988t cells treated for 72h with the indicated concentrations of JQ-1, measured by flow cytometry using DCFH-DA staining.

(O) qPCR analysis of *FASN* mRNA levels in PaTu-8988t cells treated with JQ-1 (500 nM, 72h) and with or without NAC (5 mM, 24h).

(P, Q) Luciferase reporter assays in PaTu-8988t cells. Assays were conducted under conditions of c-Myc depletion (P), overexpression (Q), or in control cells.

(R, S) KEGG pathways enrichment analysis based on GEO database (GEO accession number: GSE117890, GSE76022).

Data are presented as means ± SEM for bar graphs and representative immunoblot images were quantified from at least three independent experiments. *p < 0.05, **p < 0.01, ***p < 0.001.


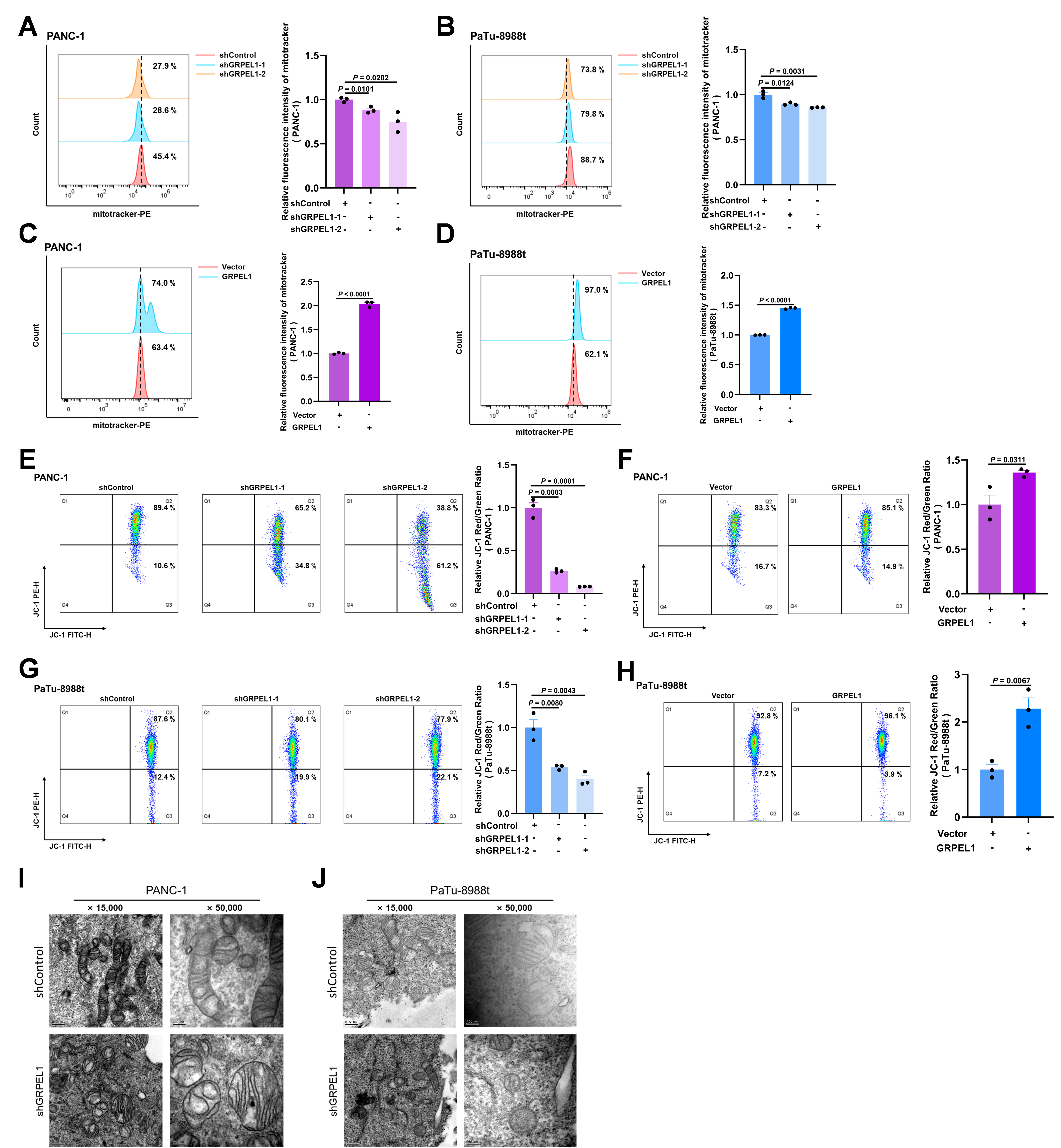


**Supplementary Figure 5.**

(A-D) Flow cytometric analysis of mitochondrial mass assessed by MitoTracker Deep Red FM staining in PANC-1 and PaTu-8988t cells with or without GRPEL1 deficiency or overexpression. Left panel: representative histograms; right panel: relative mean fluorescence intensity.

(E-H) Flow cytometric analysis of mitochondrial membrane potential assessed by JC-1 staining in PANC-1 and PaTu-8988t cells with or without GRPEL1 deficiency or overexpression. Left panel: representative histograms; right panel: relative mean fluorescence intensity.

(I, J) Mitochondrial morphology was observed by transmission electron microscopy in PDAC cell lines with or without GRPEL1 depletion. (×15,000 magnification, scale bar: 0.5 μm; ×50,000 magnification, scale bar: 200 nm.)

Data are presented as means ± SEM for bar graphs and representative immunoblot images were quantified from at least three independent experiments.


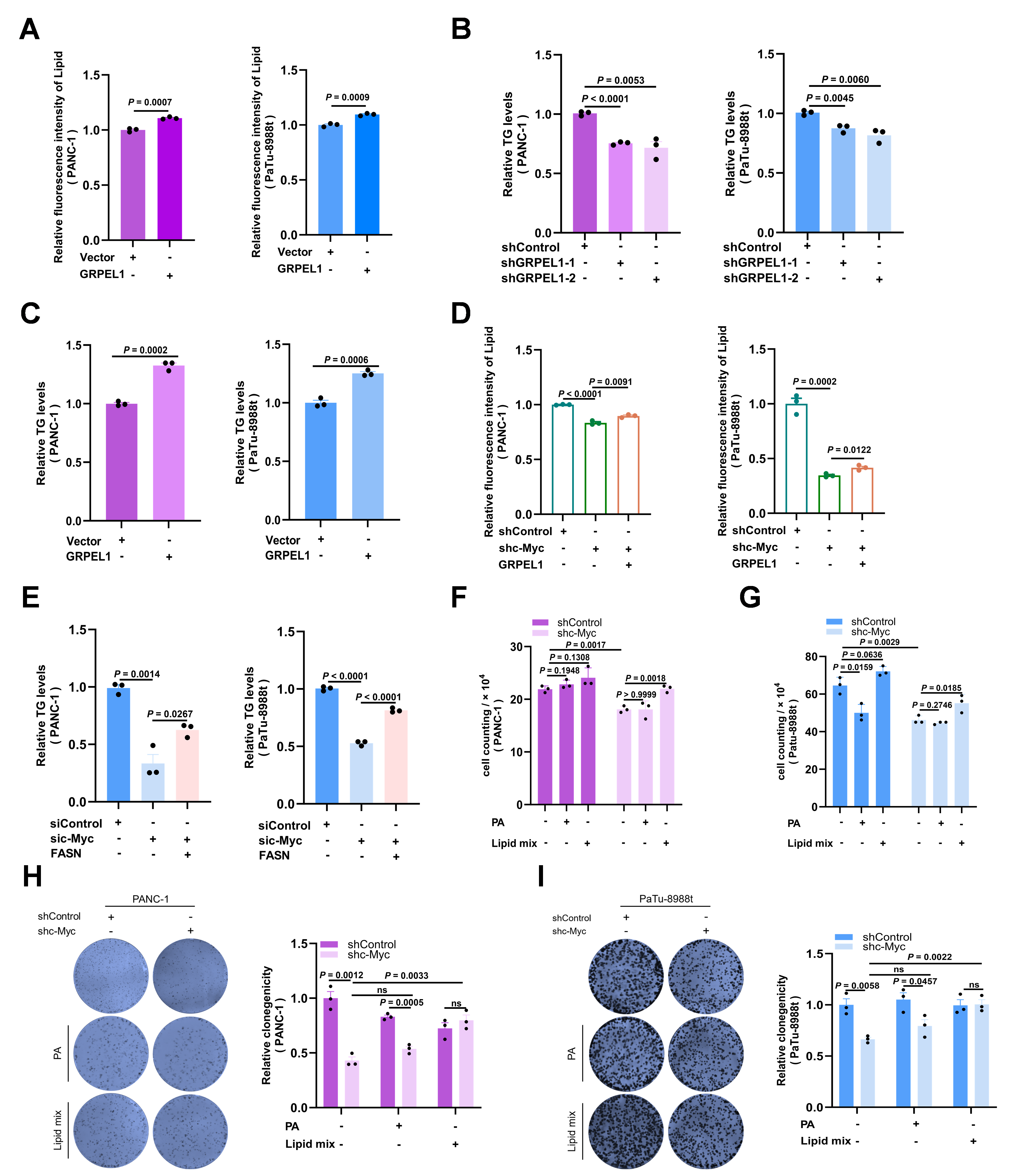


**Supplementary Figure 6.**

(A) Neutral lipid content in PANC-1 and PaTu-8988t cells with or without GRPEL1 overexpression, measured by flow cytometry using Bodipy 493/503 staining (data shown as relative MFI).

(B, C) Relative triglyceride content in PANC-1 and PaTu-8988t cells with or without GRPEL1 depletion, measured using a commercial kit.

(D) Neutral lipid content in PANC-1 and PaTu-8988t cell models, measured by flow cytometry (data shown as relative MFI).

(E) Relative triglyceride content in the indicated PANC-1 and PaTu-8988t cell models, measured using a commercial kit.

(F-I) Relative cell proliferation (F, G) and colony formation ability (H, I) of PANC-1 and PaTu-8988t cells cultured in normal medium with or without c-Myc depletion, followed by exogenous PA or lipid mix supplementation at indicated concentrations.

Data are presented as means ± SEM for bar graphs and representative immunoblot images were quantified from at least three independent experiments.


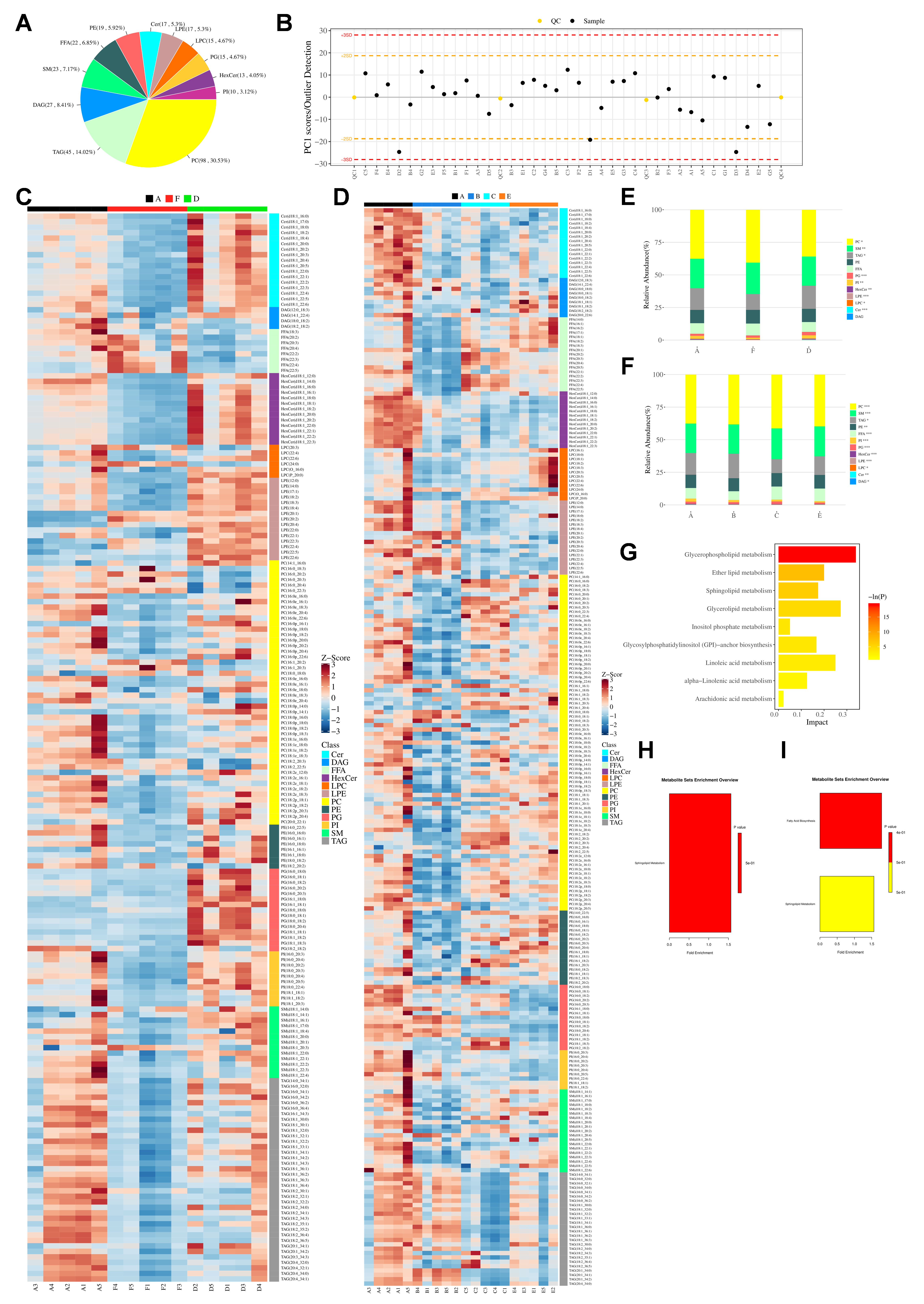


**Supplementary Figure 7.**

(A) The pie chart depicts the proportional distribution of metabolite classes based on lipidomics profiling of PaTu-8988t cells.

(B) Multivariate quality control chart was used to reflect the stability of the analytical process by monitoring the position and trends of sample scores.

(C, D) Heatmaps of differential metabolites (|Log₂FC| ≥ 0 and *P* < 0.05). Hierarchical clustering of significantly altered metabolites across all sample groups: (A) Control; (B) GRPEL1 knockdown; (C) GRPEL1 rescue; (D) c-Myc knockdown + GRPEL1 rescue; (E) GRPEL1 overexpression; (F) c-Myc knockdown. Group A serves as the common control for both panels.

(E, F) Stacked bar chart showing the median relative abundance of metabolite classes per group. Each bar represents one sample group, with segments colored according to different metabolite classes. The height of each segment corresponds to the median relative abundance of that class within the group.

(G) The chart displays significantly enriched KEGG pathways (hsa) identified from differential metabolites. Pathways are ranked by their enrichment significance, represented by the -LnP value (negative natural logarithm of the *P*-value).

(H, I) Significantly altered metabolic pathways were identified using pathway-associated metabolite sets from the Small Molecule Pathway Database.


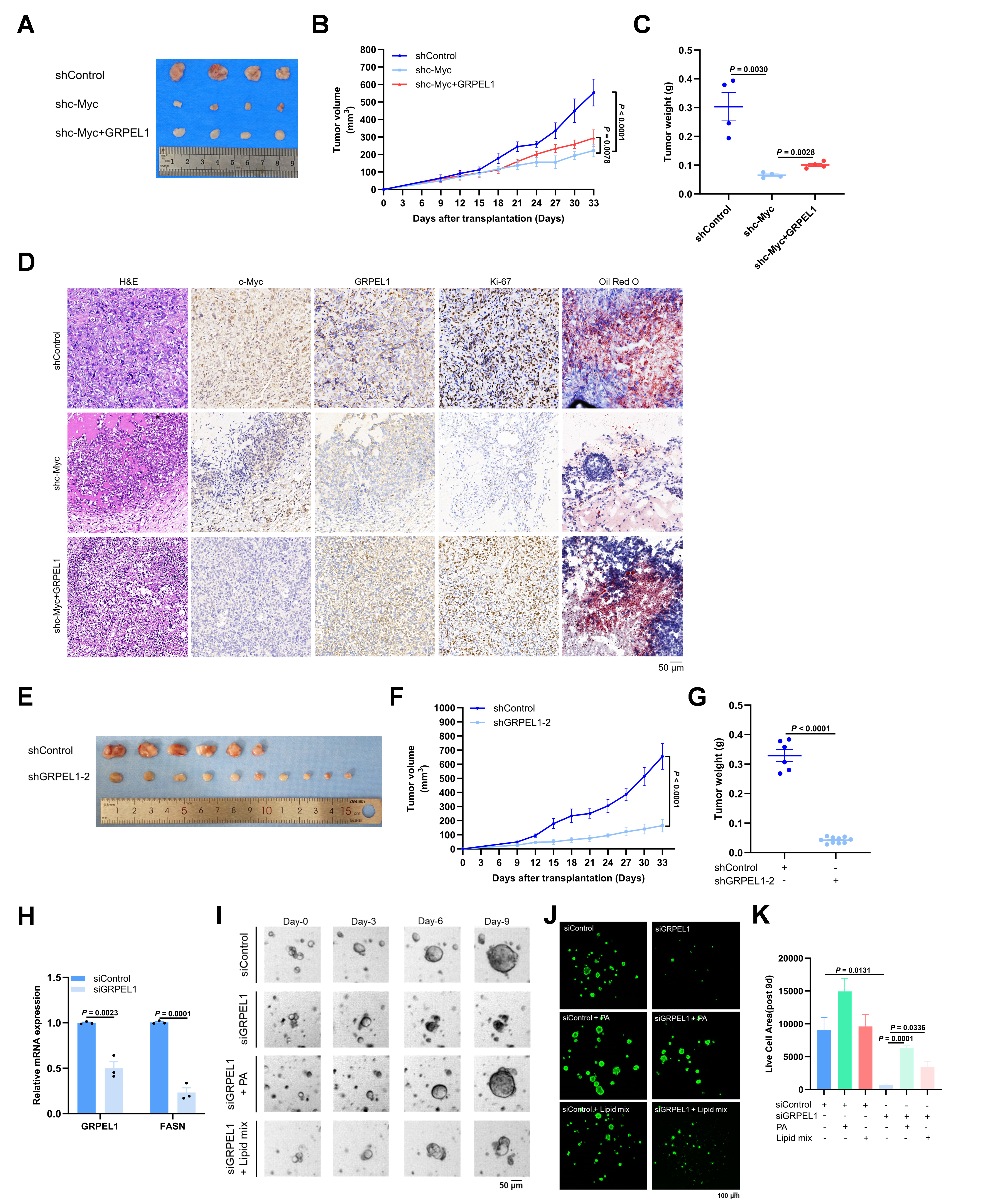


**Supplementary Figure 8.**

(A-C) Images (A), volumes (B), and weights (C) of subcutaneous PDAC tumors derived from different PaTu-8988t cell models (injected with 5 × 106 cells, N = 4).

(D) Representative images of H&E staining, c-Myc, GRPEL1 and Ki-67 IHC staining, and Oil Red O staining in different groups of xenograft tumor tissues.

(E-G) Images (E), volumes (F), and weights (G) of subcutaneous PDAC tumors derived from PaTu-8988t cells with or without GRPEL1 depletion (injected with 5 × 106 cells, N = 6 at least).

(H) The expression of GRPEL1 and FASN in PDAC organoids was detected by qPCR.

(I) Representative bright-field images show the growth of PDAC organoids with or without GRPEL1 interference and supplemented with PA (50 μM) and lipid mix (1/500) after GRPEL1 interference (N = 2).

(J, K) Calcein AM/PI staining was used to assess the cell viability of PDAC organoids with or without GRPEL1 interference and treated with or without PA or lipid mix.

Data are presented as means ± SEM for bar graphs and representative immunoblot images were quantified from at least three independent experiments.
