# Supplementary material for: c-Myc/GRPEL1 maintains fatty acid synthesis via FASN to support PDAC cell proliferation
Source: Cell Death Dis. 2026 Feb 5;17(1):205. doi: 10.1038/s41419-026-08439-0 (PMC12894993; doi:10.1038/s41419-026-08439-0)
Supplement: Supplementary file 2 — Supplementary Materials [file 41419_2026_8439_MOESM2_ESM.docx]

**Supplementary Materials:**

**Sequences of the qPCR Primers:**

| Gene | forward sequence | reverse sequence |
| --- | --- | --- |
| c-Myc | GGCTCCTGGCAAAAGGTCA | CTGCGTAGTTGTGCTGATGT |
| GRPEL1 | GCAGACACTGAGAACTTACGGC | CTGTGTTGCCTTCTCCAGAACG |
| FASN | CGCGTGGCCGGCTACTCCTAC | CGGCTGCCACACGCTCCTCT |
| GAPDH | GGCCTCCAAGGAGTAAGACC | AGGGGAGATTCAGTGTGGTG |
| GPAM | GATGTAAGCACACAAGTGAGGA | TCCGACTCATTAGGCTTTCTTTC |
| STARD7 | GTGACACCTCGGCAGTTCTT | CCACATCCCTCTCAATCACC |
| IDI1 | GCCGCAGACTGTGCTCAAAGC | CCTGTTGCTTGTCGAGGTGGTT |
| SFXN1 | ACCAGTCCTTCAATGCCGTCGT | GAGTCCTAGAGCTGTTGCTACG |
| SFXN3 | GGGTGAATTGCCTTTAGACATCA | GCAGCAGATTTCGAGGATCAGT |

**siRNA sequences:**

| Gene | forward sequence | reverse sequence |
| --- | --- | --- |
| sic-Myc-1 | CGAUGUUGUUUCUGUGGAATT | UUCCACAGAAACAACAUCGTT |
| sic-Myc-2 | CCAAGGUAGUUAUCCUUAATT | UUAAGGAUAACUACCUUGGTT |
| siGRPEL1-1 | GGCAGACACUGAGAACUUATT | UAAGUUCUCAGUGUCUGCCTT |
| siGRPEL1-2 | GAAGGUGUUCACAAAGCAUTT | AUGCUUUGUGAACACCUUCTT |

**Sequences of the CHIP-qPCR Primers:**

| Gene | forward sequence | reverse sequence |
| --- | --- | --- |
| c-Myc | GGCAGGGACTCTAAGTCTGG | ATTGGGGCACAACTAAAGC |

**Clinical Data of the PDAC TMA Cohort:**

(The finalized tissue microarray, confirmed by an experienced pathologist, included 67 PDAC specimens and their matched paracancerous tissues. The relevant clinical information is presented below.)
