# Supplementary material for: c-Myc/GRPEL1 maintains fatty acid synthesis via FASN to support PDAC cell proliferation
Source: Cell Death Dis. 2026 Feb 5;17(1):205. doi: 10.1038/s41419-026-08439-0 (PMC12894993; doi:10.1038/s41419-026-08439-0)
Supplement: Supplementary file 3 — westblot original figure [file 41419_2026_8439_MOESM3_ESM.pdf]

Figure 1 H

PaTu-8988t

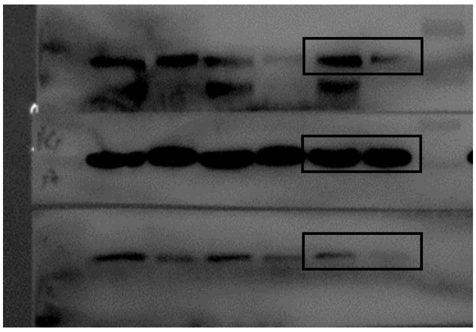

c-Myc

GAPDH

GRPEL1

PANC-1

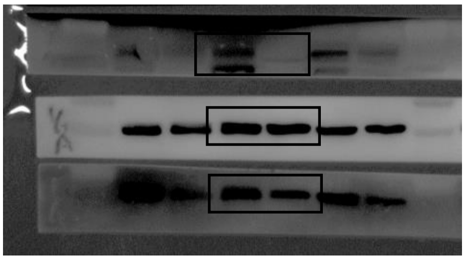

c-Myc

GAPDH

GRPEL1

HEK-293T

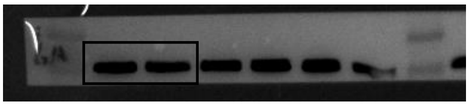

GAPDH

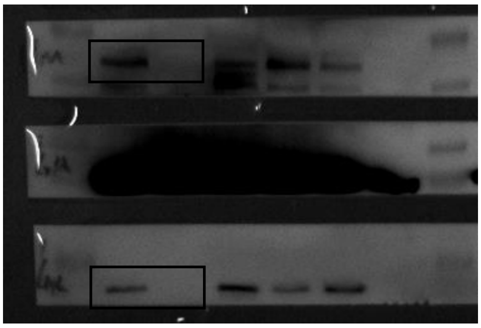

c-Myc

GRPEL1

Figure 1 I

HEK-293T

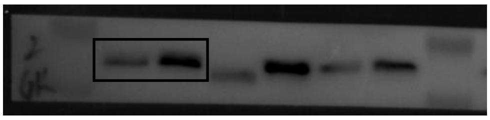

GRPEL1

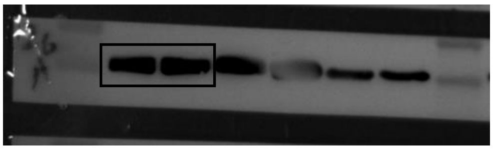

GAPDH

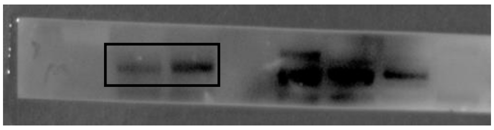

c-Myc

PANC-1

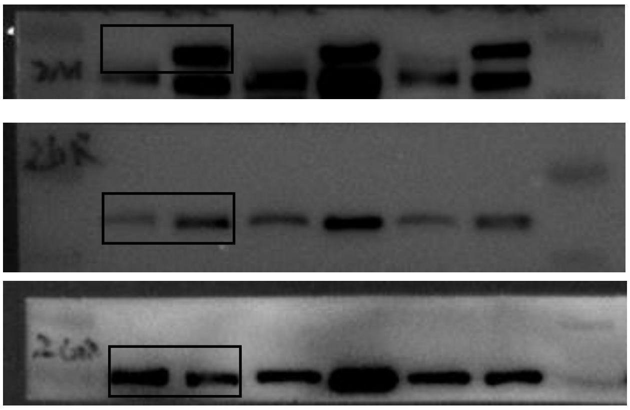

c-Myc

GRPEL1

GAPDH

PaTu-8988t

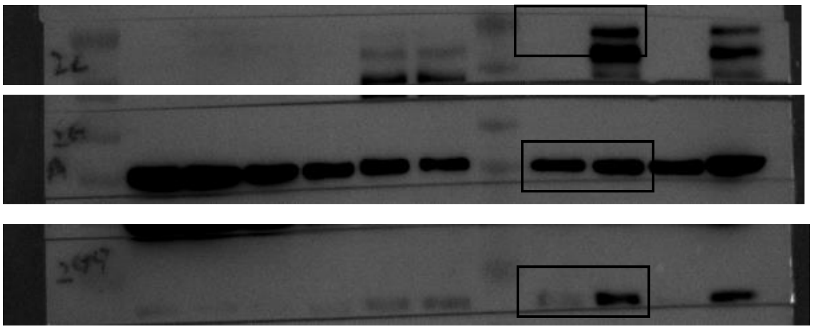

c-Myc

GAPDH

GRPEL1

Figure 1 J

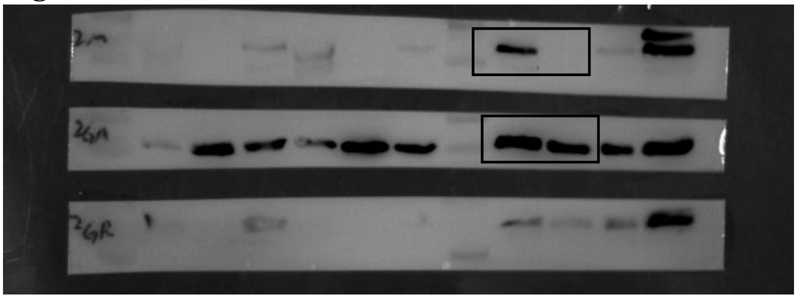

c-Myc

GAPDH

Figure 1 K

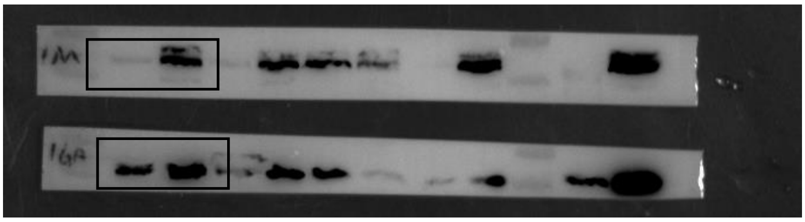

c-Myc

GAPDH

Figure 2 A

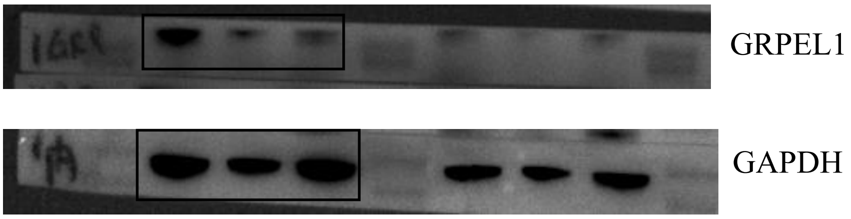

Figure 2 B

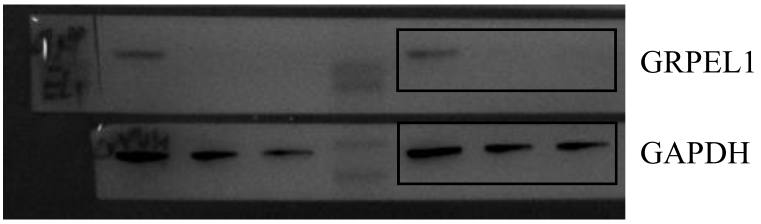

Figure 2 K

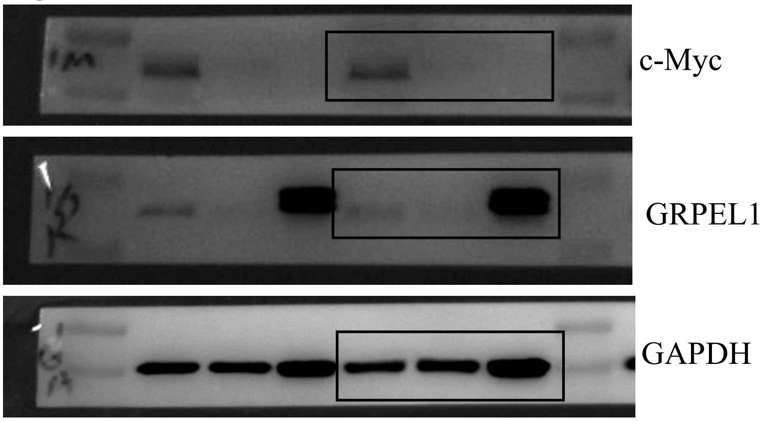

Figure 2 N

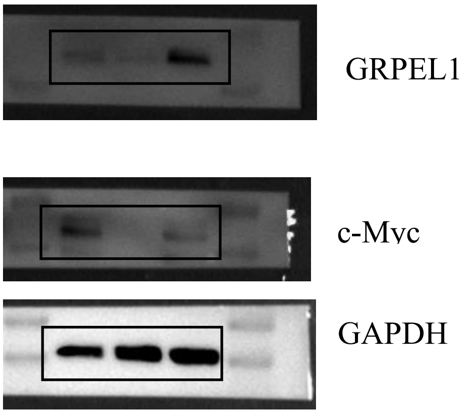

Supplementary Figure 2 A

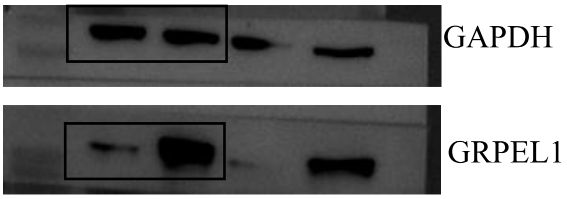

Supplementary Figure 2 B

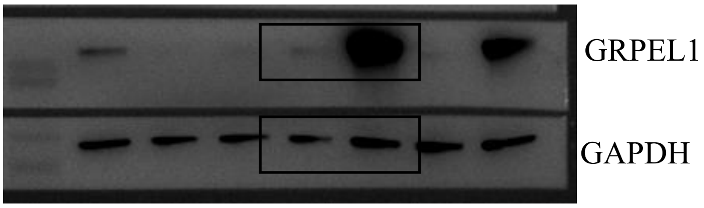

Figure 3 F

PANC-1

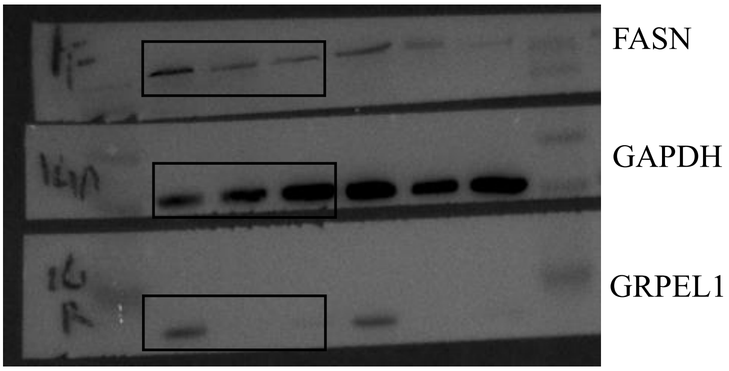

PaTu-8988t

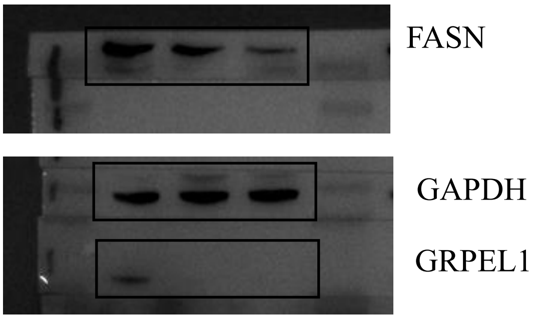

Figure 3 I

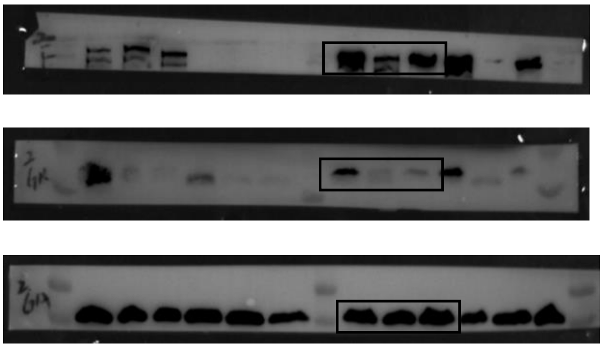

Figure 3 L

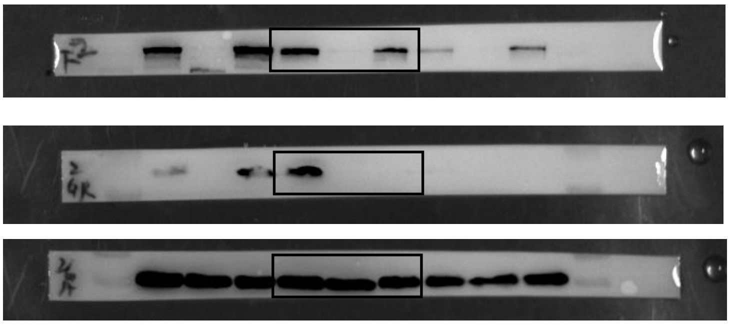

Figure 4 K

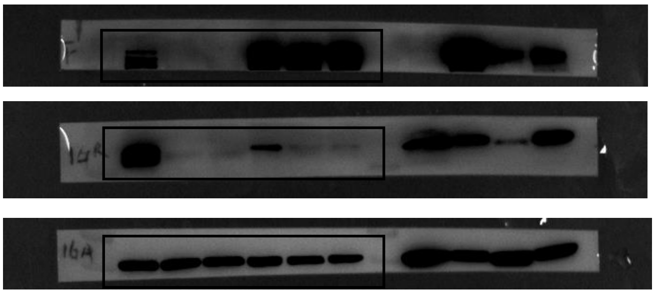

Figure 4 N

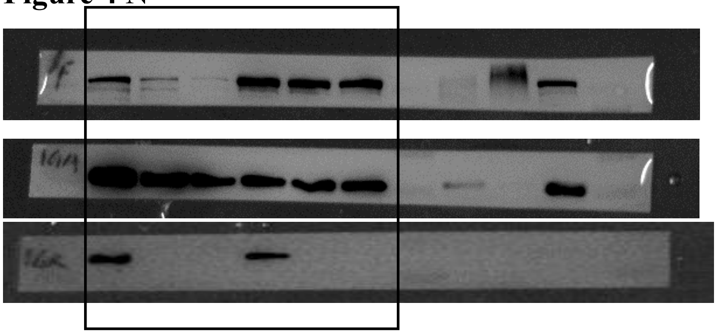

Supplementary Figure 4 I

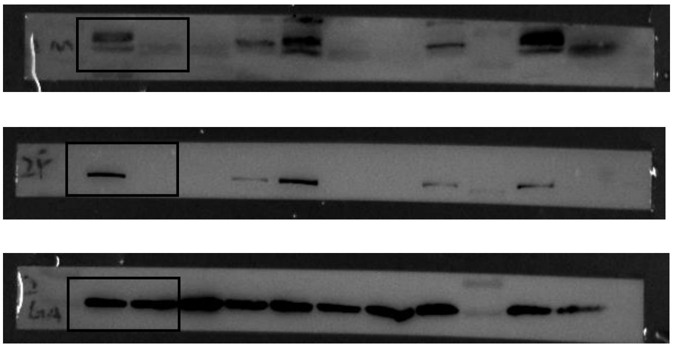

c-Myc

FASN

GAPDH

Supplementary Figure 4 J

1 2

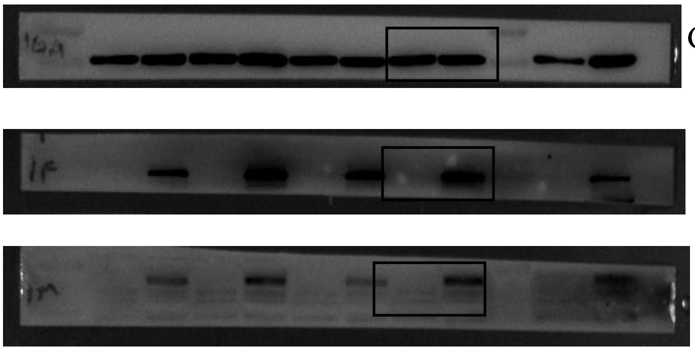

GAPDH (note: lane 1:sic-Myc; lane 2: siControl)

FASN

c-Myc

Supplementary Figure 4 K

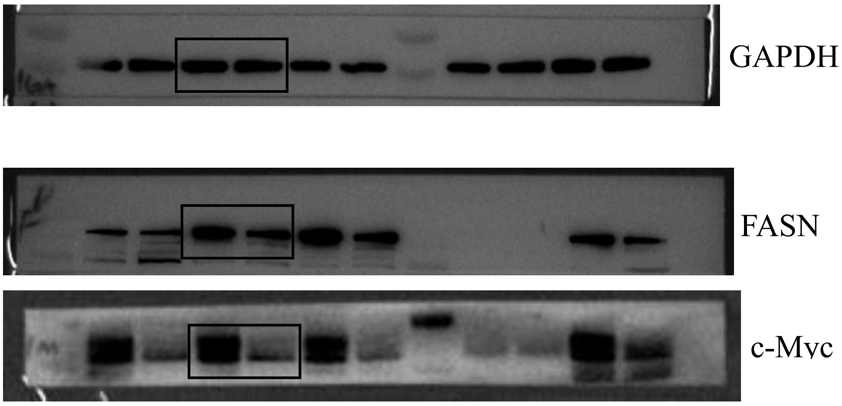

GAPDH

FASN

c-Myc

Supplementary Figure 4 L

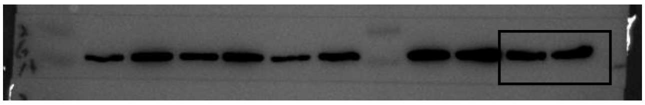

GAPDH

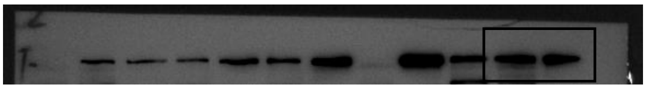

FASN

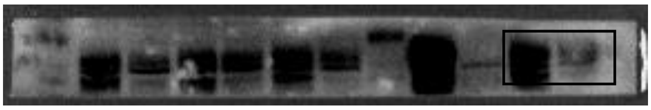

c-Myc

Supplementary Figure 3 B
PANC-1

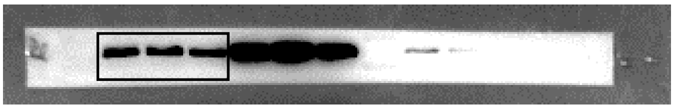

P-eIF2α

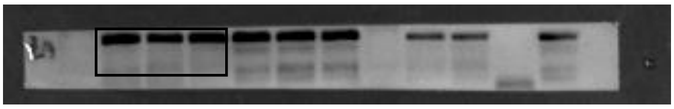

eIF2α
(stripping)

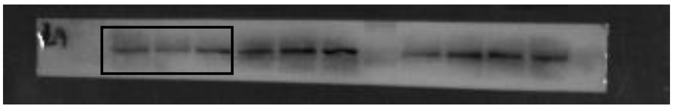

ATF4

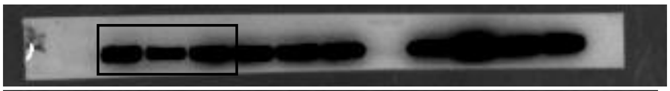

GAPDH
(stripping)

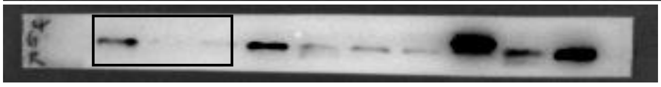

GRPEL1
(same sample, representative technical replicate)

PaTu-8988t

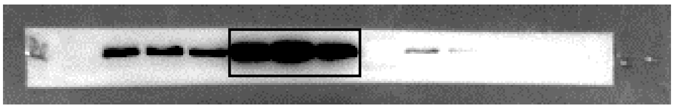

P-eIF2α

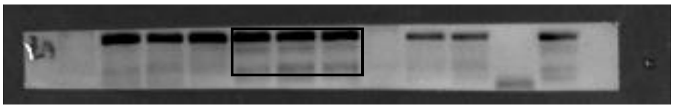

eIF2α
(stripping)

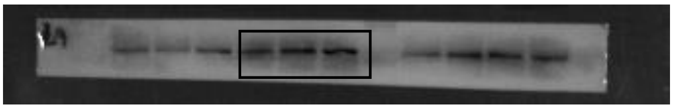

ATF4

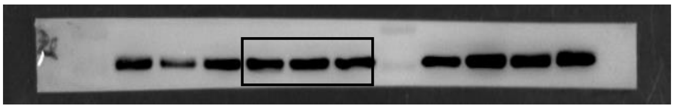

GAPDH
(stripping)

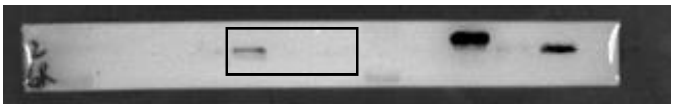

GRPEL1

Supplementary Figure 3 C

PANC-1

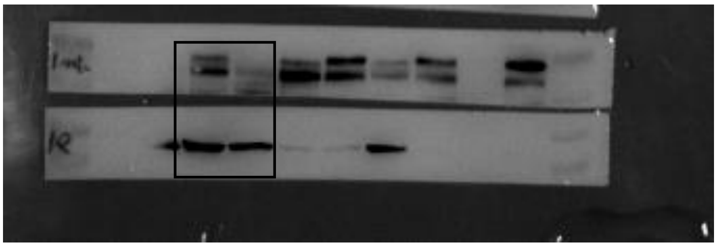

c-Myc

P-eIF2α

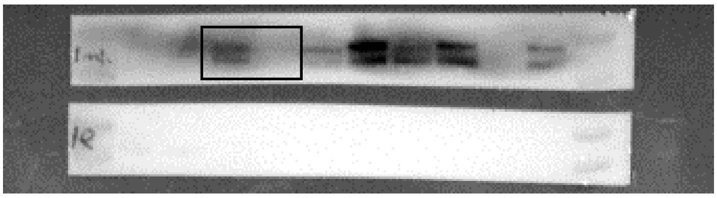

ATF4
(stripping)

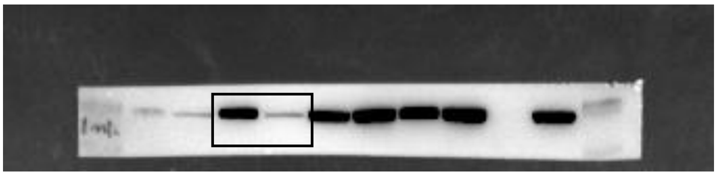

eIF2α
(stripping)

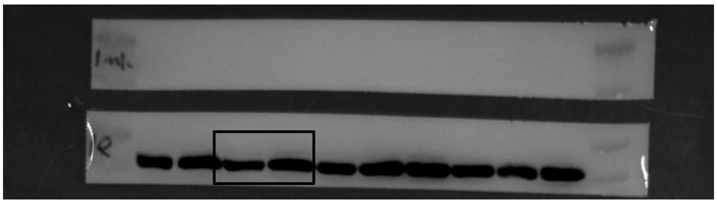

GAPDH
(stripping)

PaTu-8988t

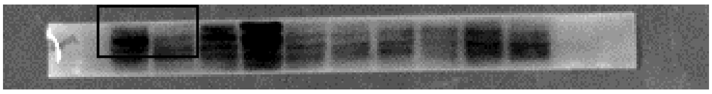

c-Myc

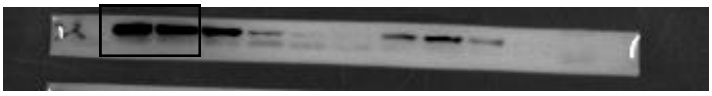

P-eIF2α

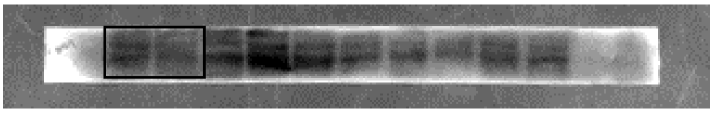

ATF4
(stripping)

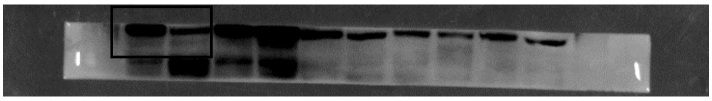

eIF2α
(stripping)

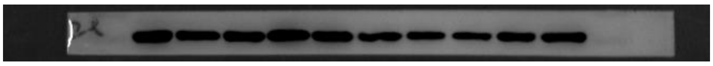

GAPDH
(stripping)

Supplementary Figure 3 D
PANC-1

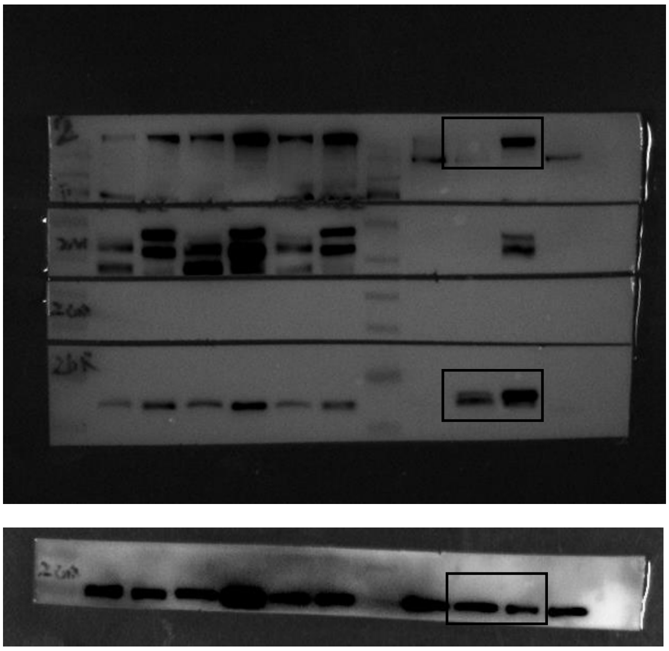

FASN

GRPEL1

GAPDH

PaTu-8988t

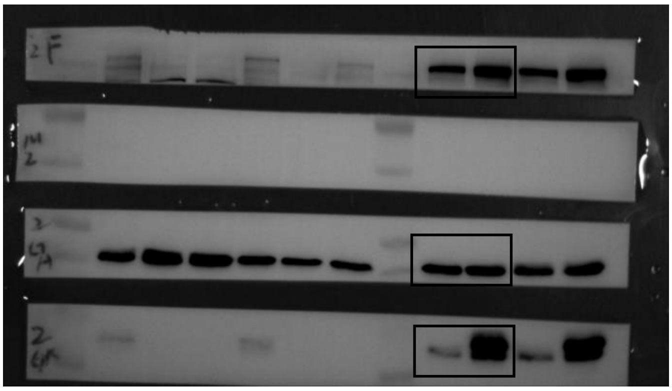

FASN

GAPDH

GRPEL1
